# New pharmacodynamic parameters linked with ibrutinib responses in chronic lymphocytic leukemia: Prospective study in real-world patients and mathematical modeling

**DOI:** 10.1371/journal.pmed.1004430

**Published:** 2024-07-22

**Authors:** Sarah Cadot, Chloe Audebert, Charlotte Dion, Soleakhena Ken, Loic Dupré, Laetitia Largeaud, Camille Laurent, Loic Ysebaert, Fabien Crauste, Anne Quillet-Mary

**Affiliations:** 1 INSERM UMR1037, CNRS UMR5071, Université Toulouse III-Paul Sabatier, Centre de Recherches en Cancérologie de Toulouse, Toulouse, France; 2 Laboratoire d’Excellence ’TOUCAN-2’, Toulouse, France; 3 Sorbonne Université, CNRS, Université Paris Cité, Laboratoire Jacques-Louis Lions UMR 7598, Paris, France; 4 Sorbonne Université, CNRS, Institut de Biologie Paris-Seine, Laboratoire de Biologie Computationnelle et Quantitative UMR 7238, Paris, France; 5 Sorbonne Université, UMR CNRS 8001, LPSM, Paris, France; 6 Institut Claudius Regaud- Institut Universitaire du Cancer de Toulouse-Oncopole, Toulouse, France; 7 Institut Toulousain des Maladies Infectieuses et Inflammatoires, INSERM, CNRS, Université Toulouse III-Paul Sabatier, Toulouse, France; 8 Department of Dermatology, Medical University of Vienna, Vienna, Austria; 9 Institut Universitaire du Cancer-Oncopole de Toulouse, Toulouse, France; 10 Université Paris Cité, CNRS, MAP5 UMR 8145, Paris, France; Washington University in St Louis, UNITED STATES OF AMERICA

## Abstract

**Background:**

One of the first clinical observations of ibrutinib activity in the treatment of chronic lymphocytic leukemia (CLL) is a rapid decline in lymph nodes size. This phenomenon is accompanied by an hyperlymphocytosis, either transient or prolonged, which is associated with distinct clinical responses and thus has an impact on long-term outcomes. Understanding which factors determine distinct disease courses upon ibrutinib treatment remains a scientific challenge.

**Methods and findings:**

From 2016 to 2021, we conducted a longitudinal and observational study in 2 cohorts of patients with chronic lymphocytic leukemia (CLL) (cohort 1, *n* = 41; cohort 2, *n* = 81). These cohorts reflect the well-known clinical features of CLL patients, such as Male/Female sex ratio of 2/1, a median age of 70 years at diagnosis, and include patients in first-line therapy (27%) or relapsed/refractory patients (73%). Blood cell counts were followed for each patient during 2 years of ibrutinib treatment. In addition, immunophenotyping and whole-body magnetic resonance imaging (MRI) were assessed in patients from cohort 1. These data were integrated in a newly built mathematical model, inspired by previous mathematical works on CLL treatment and combining dynamical and statistical models, leading to the identification of biological mechanisms associated with the 2 types of clinical responses. This multidisciplinary approach allowed to identify baseline parameters that dictated lymphocytes kinetics upon ibrutinib treatment. Indeed, ibrutinib-induced lymphocytosis defined 2 CLL patient subgroups, transient hyperlymphocytosis (tHL) or prolonged hyperlymphocytosis (pHL), that can be discriminated, before the treatment, by absolute counts of CD4^+^ T lymphocytes (*p* = 0.026) and regulatory CD4 T cells (*p* = 0.007), programmed cell death protein 1 PD1 (*p* = 0.022) and CD69 (*p* = 0.03) expression on B leukemic cells, CD19/CD5^high^/CXCR4^low^ level (*p* = 0.04), and lymph node cellularity. We also pinpointed that the group of patients identified by the transient hyperlymphocytosis has lower duration response and a poor clinical outcome. The mathematical approach led to the reproduction of patient-specific dynamics and the estimation of associated patient-specific biological parameters, and highlighted that the differences between the 2 groups were mainly due to the production of leukemic B cells in lymph node compartments, and to a lesser extent to T lymphocytes and leukemic B cell egress into bloodstream. Access to additional data, especially longitudinal MRI data, could strengthen the conclusions regarding leukemic B cell dynamics in lymph nodes and the relevance of 2 distinct groups of patients.

**Conclusions:**

Altogether, our multidisciplinary study provides a better understanding of ibrutinib response and highlights new pharmacodynamic parameters before and along ibrutinib treatment. Since our results highlight a reduced duration response and outcome in patients with transient hyperlymphocytosis, our approach provides support for managing ibrutinib therapy after 3 months of treatment.

**Trial registration:**

ClinicalTrials.gov NCT02824159.

## Abstract 2

Anne Quillet-Mary and team combine clinical parameters, biological monitoring, whole blood tissue imaging and mathematical modeling to identify key biological and pharmacodynamic factors involved in ibrutinib response in chronic lymphocytic leukemia.

## Author summary

**Why was this study done?:**

- Treatment of chronic lymphocytic leukemia (CLL) was revolutionized by the introduction of targeted therapies such as ibrutinib. Previous studies showed that lymphocyte kinetics under ibrutinib treatment are related to clinical outcome, but some potential mechanisms underlying these responses have not been fully explored.
- This study was done to decipher key biological and pharmacodynamic factors involved in ibrutinib response.

**What did the researchers do and find?:**

- Relying on a cohort of patients treated with ibrutinib, we combined clinical parameters, biological monitoring, whole-body tissue imaging, and mathematical modeling to decipher key biological and pharmacodynamic factors underlying the 2 distinct groups of ibrutinib response, transient or prolonged hyperlymphocytosis.
- We elaborated a mathematical model, based on previous ones of CLL and combining both dynamical and statistical approaches, that characterizes inter-patient variability and estimates patient-specific biological parameters associated with the 2 groups of response.
- We identified clinical factors (age, absolute lymphocyte counts) and biological factors, such as absolute counts of CD4^+^ T lymphocytes and regulatory CD4 T cells, programmed cell death protein 1 and CD69 expression on B leukemic cells, CD19/CD5^high^/CXCR4^low^ level in blood, and lymph node cellularity, before and during ibrutinib therapy. These biomarkers are predictive of ibrutinib response.
- The mathematical approach confirmed the identified clinical factors, by highlighting that the differences between the 2 types of response to ibrutinib treatment were mainly due to the production of leukemic B cells in lymph node compartments, and to a lesser extent to T lymphocytes and leukemic B cell egress into bloodstream.
- We found that the group of patients identified by the transient hyperlymphocytosis has lower duration response and a poor clinical outcome.

**What do these findings means?:**

- Estimation of patient-specific biological parameters in the mathematical models highlights that only few biological processes are group-specific when CLL patients are treated with ibrutinib.
- Biomarkers defined in this study can be easily monitored in CLL patients before and during ibrutinib treatment.
- These results offer potential support for optimizing the management of ibrutinib treatment, especially in the first months of therapy.
- Access to additional data, especially longitudinal magnetic resonance imaging (MRI) data, could strengthen the conclusions regarding leukemic B cell dynamics in lymph nodes.

## Introduction

Management of chronic lymphocytic leukemia (CLL), in first-line and relapsed/refractory settings, has been revolutionized by the introduction of oral, targeted agents against Bruton Tyrosine Kinase (ibrutinib), PI-3Kinase (idelalisib), and Bcl-2 (venetoclax) [1,2]. In real-world practice, ibrutinib has been used since 2014 in relapsed/refractory patients and in first-line patients with TP53 abnormalities. The mechanisms by which ibrutinib targets CLL and restores normal immune subsets include: direct cell killing (mostly in tissues), egress from tissue niches, and inhibition of proliferation by targeting B cell receptor signaling and activation of canonical NF-kB pathway, primarily in the lymph node (LN) tumor microenvironment [3,4]. These effects led to an increase of absolute lymphocyte counts (ALCs) in parallel of efflux from tissue compartments, whose kinetics appears heterogeneous across patients’ cohorts [5–8].

Kinetics of lymphocytosis upon ibrutinib treatment has been first described by Wiestner’s group [6]. In this Phase 2 study [6], 3 profiles of ALCs were described in which 1 subset showed a long-lasting ALCs increase over time (prolonged hyperlymphocytosis (pHL), linked to lower baseline ALCs, immunoglobulin heavy chain variable region genes mutated status, and bulky disease). Importantly, none of those subsets were found correlated to clinical outcomes. Prolonged hyperlymphocytosis with a slight modification of definition was reported in 69% of RESONATE (relapsed/refractory CLL) and 57% of RESONATE-2 (first-line CLL without deletion 17p) patients respectively, but this time was found to be correlated with an improvement of progression-free survival (PFS) only in the relapsed/refractory patients after 19 months follow-up [8,9]. It suggested that lack of initial hyperlymphocytosis could be considered as an easy and rapidly assessable risk factor for progressions [8,10].

Besides its efficacy against CLL cells, ibrutinib displays “on-target” and “off-tumor” effects, explaining some of the adverse events [11,12], but might also be responsible for disease-related immune suppression state [13–15]. One of the hallmarks of this disease is indeed a profound immunosuppression due to impairment of T cells proliferation, cytokine production, immune synapse formation, leading to accumulation of exhausted programmed cell death protein 1 (PD1^+^) T cells, and increase in CD4^+^CD25^high^CD127^low^ regulatory T cells (Tregs) [15]. Under ibrutinib, immuno-monitoring of PD1 levels at the surface of CD4^+^ and CD8^+^ T cells have been reported from small cohorts (7, 14, and 17 patients) in 3 independent studies, without correlation to clinical outcomes [16–18]. On the leukemic B cell compartment, ibrutinib treatment has been shown to be associated with a reduction of PD1 expression (more pronounced than on the T cell compartment) [18], the consequences of which remain unknown.

Mathematical modeling contributed to better understand ibrutinib-induced mechanisms in CLL patients [5]. The model introduced by Wodarz and colleagues [5] and later used in Burger and colleagues [7] consisted in 2 ordinary differential equations (ODEs) describing leukemic B cell dynamics in LN/spleen and blood. The authors concluded that ibrutinib mainly affects leukemic B cell death, with larger death rates in tissues than in blood. In 2014, Komarova and colleagues [19] focused on the development of ibrutinib resistance using methods from evolutionary and computational biology. These works impacted the clinical understanding of CLL and have been essential in the development of current CLL treatments.

Here, using a longitudinal and observational study of CLL patients, we investigated clinico-biological and imaging parameters predicting 2 groups of response induced by ibrutinib (ALCs but also specific immunomodulatory effects of ibrutinib). We explored whether these markers could be correlated to each other and/or to duration of treatment response. To do so, we have designed a mathematical model built on our data and previous mathematical models [5,7] and focused on both leukemic B and T cell counts co-evolution. We have elaborated a modeling approach based on the coupling of ODE describing leukemic B and T cell dynamics and nonlinear mixed effect (NLME) modeling. This approach enables to describe observed inter-patient variability. To our knowledge, no NLME model has been studied for CLL under treatment. We combined the results of clinical analyses and mathematical model parameter estimation to identify which effects explain the 2 ibrutinib-induced patterns of lymphocytosis.

## Methods and materials

### Ethics statement, patients, and treatment

Peripheral blood samples from ibrutinib-treated CLL patient (*n* = 122: cohort 1, *n* = 41; cohort 2, *n* = 81) were obtained from the Hematology Department with written informed consent and referenced in INSERM cell bank. According to French law, INSERM cell bank has been registered with the Ministry of Higher Education and Research (DC-2013-1903). Clinical and biological annotations of the samples have been reported to the Comité National Informatique et Liberté. This longitudinal and observational study was approved by the competent authority (ANSM, n° 1551668A-11), the ethics committee (N° CPP16-004a) and registered by Clinical-Trials.gov (NCT02824159).

This study is reported as per the Strengthening the Reporting of Observational studies in Epidemiology (STROBE) guideline (S1 STROBE Checklist).

### Immuno-monitoring study design

Among 122 ibrutinib-treated CLL patients included in the protocol, 41 patients were followed for a two-year period from the treatment initiation (M0: Month 0). Peripheral blood samples were collected at different times of the treatment phase (M0, M1, M2, M3, M6, M12, M18, and M24).

#### Immunofluorescence staining and analysis

For cell surface staining, peripheral blood mononuclear cells (PBMCs) were incubated with conjugated antibodies (S1 Table) in phosphate buffer saline with 1% inactivated fetal calf serum for 20 min at 4°C in the dark. For intra cellular staining, surface labeled PBMC were fixed for 15 min at room temperature in paraformaldehyde 1.4%, then permeabilized using BD Phosflow Perm Buffer II (BD Bioscience), washed and stained with BTK (Bruton Tyrosine Kinase) and p-BTK conjugated-antibodies for 20 min at 4°C in the dark. Samples were measured on a BD LSR II cytometer and analyzed with BD FACS Diva software (BD Bioscience).

#### In vitro blood cell depletion assay

In vitro ibrutinib sensitivity was quantified using B cell depletion assay [20]. Briefly at each time of ibrutinib treatment, fresh PBMC were seeded at 1 × 10^7^ cells/ml in culture medium (providing long-term viability) and treated by relevant doses of ibrutinib for 7 days. CD19^+^/CD5^+^ (B leukemic cells) levels were determined by flow cytometry. B-cell depletion relative to untreated controls was determined by flow cytometry combined with absolute number quantification as previously described [20].

#### Statistical analyses of medical and biological data

They were done using two-tailed Mann–Whitney test (unpaired samples) or Student *t* test (paired samples).

### Lymph nodes segmentations

Whole body anatomic magnetic resonance imaging (MRI) was acquired at baseline (M0) for 26 patients of cohort 1 (who accepted MRI protocol) and consisted on several 3D volume stages of T1 weighted axial images. These patients who have been analyzed with diffusion-weighted MRI study were the same patients analyzed in our previous study [21] except 1 patient whose acquisition encountered problem of image reconstruction. The parameters of the diffusion-weighted MRI are the following: Repetition Time/Echo Time/Inversion Time = 6,930 ms/71 ms/160 ms with 2 b-value of 50 and 800 s/mm^2^. Diffusion images were obtained by averaging over 2 acquisitions. The number of stages was adjusted according to patient size to cover the whole body. To reduce motion artifact, volume stages corresponding to thoracic and abdominal regions were acquired in breath holding condition. Cervical, axillary, mediastinal, retroperitoneal, iliac, inguinal, and splenic regions of interest were segmented with a dedicated semiautomatic method [21]. After a clinical validation by 2 radiologists, the lymph nodes segmentations allowed us to compute the volumes (in ml) and to map these regions of interest on the diffusion modality from which the apparent diffusion coefficient (ADC) (mm^2^/s) was done for the first 9 patients. ADC is related to cellularity that captures the degree of abnormal cellular packing and density.

### Cell count dynamics study and modeling

Among the 122 patients, group classification was defined according to previously described clinical results [8,9] and thus, pHL was defined as having an ALCs at M3 larger than at M0 (ALC M3 > ALC M0).

Based on known interactions between B and T cells, and inspired by Wodarz and colleagues [5] model, a generic model of B and T cell dynamics under ibrutinib treatment was built and validated on medical data to reproduce the behavior of an average patient. The model accounts for clinical observable variables: CD19^+^/CD5^+^ B cell counts in LN (denoted by *B*_*LN*_), and CD19^+^/CD5^+^ B cell (*B*_*bl*_), CD4 T cell (*T*_4_), CD8 T cell (*T*_8_), Natural Killer (NK) cell (*T*_*NK*_), and Regulatory CD4 T cell (*T*_*regs*_) counts in blood. Dynamics of leukemic B cells are described by a model similar to the one in Wodarz and colleagues [5], where cells exit LN with a rate *F*_*out*_ and are produced within LN with a constant rate *F*_*in*_. Dynamics of T cells follow a standard equation, including T cell renewal and production from LN. Model’s parameters are described in Table 1 and details of the modeling procedure are available in S1 File Modeling.

**Table 1:** Variables and parameters of the model of B and T cell dynamics. **(**d–day; LN, lymph nodes; NK, Natural Killer cells; Tregs, regulatory CD4 T cells).

*Variables and parameters*	*Unit*	*Description*
*B*_*LN*_	cell count	Leukemic B cell count in LN
*B*_*bl*_	cell count	Leukemic B cell count in blood
*T*_4_	cell count	CD4 T cell count in blood
*T*_8_	cell count	CD8 T cell count in blood
*T*_*NK*_	cell count	NK cell count in blood
*T*_*regs*_	cell count	Tregs cell count in blood
$B_{LN}^{0}$	cell count	Initial leukemic B cell count in LN
$B_{bl}^{0}$	cell count	Initial leukemic B cell count in blood
$T_{4}^{0}$	cell count	Initial CD4 T cell count in blood
$T_{8}^{0}$	cell count	Initial CD8 T cell count in blood
$T_{NK}^{0}$	cell count	Initial NK cell count in blood
$T_{regs}^{0}$	cell count	Initial Tregs cell count in blood
*F*_*out*_	d^-1^	Lymph node exit rate
*F*_*in*_	cell.d^-1^	Leukemic B cell lymph node production rate
*μ*_*B*_	d^-1^	Leukemic B cell death rate
*μ*_4_	d^-1^	CD4 T cell death rate
*μ*_8_	d^-1^	CD8 T cell death rate
*μ*_*NK*_	d^-1^	NK cell death rate
*μ*_*reg*_	d^-1^	Tregs death rate
*t*	d	Time

The model has been validated, in patients with and without pHL, separately by data fitting (mean values of cell counts at M1, M2, M3, M6, M12, M18, and M24 for blood measurements, and M1, M12 an M24 for LN measurements, obtained through volume estimations MRI measurements). Least-squares minimization has been used to optimize parameter values. Parameter value estimation and data fitting have been performed using Data2Dynamics [22,23], a Matlab R2019b add-on (S1 File Modeling), and for each parameter, profile likelihood-based confidence intervals [24] are provided.

Model development and its analysis have been performed following Garnett and colleagues guidelines [25].

### Population approach and inter-patient variability

To account for inter-patient variability in the mathematical model, we used a population approach based on mixed-effect modeling [26]. NLME models allow the description of inter-patient variability within a population of individuals by assuming all individuals in the population (here CLL patients) share common characteristics (fixed effects) while each patient is unique and differs from the average behavior by a specific value (random effect) [26–29]. Details are available in S1 File Modeling.

Parameter values were estimated with Stochastic Approximation Expectation-Maximization algorithm [30]. A categorical covariate was used to characterize groups of patients. Clinical cell counts were pooled together, and then parameter values were estimated assuming that fixed effects were different between groups of patients. Estimated covariate parameters were tested to be significantly different from zero with a Wald test implemented in Monolix software [30] and a *p*-value threshold at 0.05 (see S1 File Modeling).

### Model selection

The generic model of B and T cell count dynamics has been selected using a procedure of model selection based on data fitting, parameter estimation, and comparison of statistical indicators (corrected Akaike information criterion). All details and tested models are provided in S1 File Modeling.

To describe inter-patient variability, an error model must be assessed for each variable, parameter correlations, and significant covariates must be selected. The Stochastic Approximation for Model Building Algorithm (SAMBA) [31] is an iterative procedure that allows to build a covariate, a correlation, and an error model automatically. The procedure relies on Monolix simulations and uses the corrected Bayesian information criterion to validate its selection.

An appropriate NLME model was selected using the SAMBA implementation in the function *buildmlx* and the variability model (random effects needed to explain the data) was built with *buildVar* function both of the R package Rsmlx [32]. SAMBA does not guarantee that a global minimum will be reached. Consequently, SAMBA was performed with 5 different initial configurations (S2 Table) and the model with all common features was selected. Finally, a multi-start approach available in Monolix [30] was performed to ensure that estimations were robust and the model was practically identifiable.

## Results

### Ibrutinib-induced lymphocytosis defines 2 CLL patient subgroups

**Fig 1:**
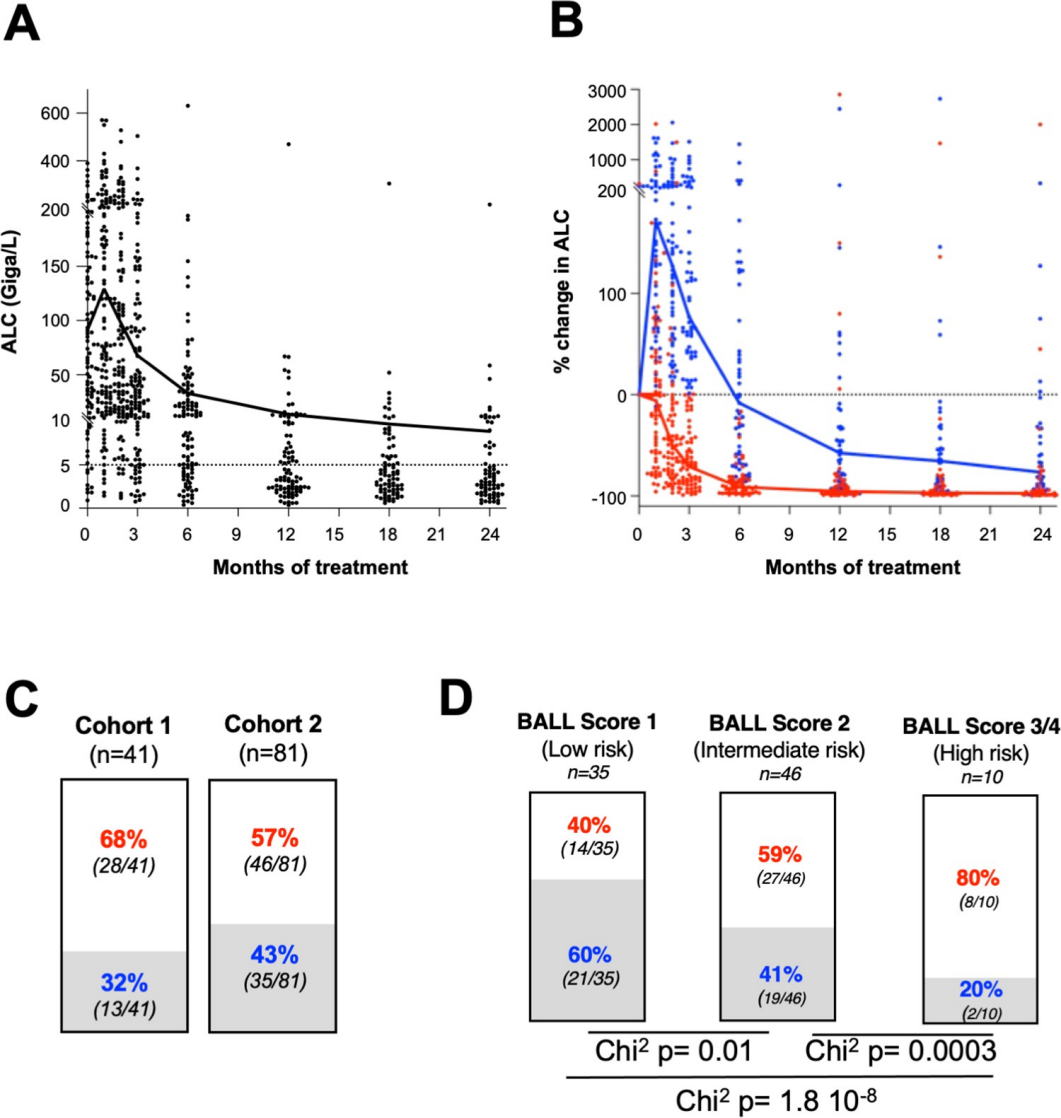
Ibrutinib-induced lymphocytosis. **(A**) ALC in cohort 1 (dotted line represents CLL ALC at diagnosis; black dots represent each patient at different time point; solid black lie: median). **(B)** Median of percent change in ALC compared to baseline in tHL (red line) and pHL (blue line) groups; red dots (tHL group) and blue dots (pHL group) represent each patient at different time point. **(C)** Percentage of tHL (red) and pHL (blue) groups in the 2 independent cohorts. **(D)** Percentage of tHL (red) and pHL groups (blue) according to the BALL score: low risk according to the BALL score equals a better overall survival prognosis based on 4 markers (ß2-microglobulin, Anemia, Lactacte Deshydrogenase, Last therapy) in the setting of relapsed/refractory receiving chemo-immunotherapy or targeted therapy [33]. ALC, absolute lymphocyte count; CLL, chronic lymphocytic leukemia; pHL, prolonged hyperlymphocytosis; tHL, transient hyperlymphocytosis.

In our 122 patients (S3 Table) treatment-induced lymphocytosis was highly variable between patients (Fig 1A). Yet, we applied the previously described cut-off [8,9] to easily classify patients according to lymphocytosis status, irrespectively of percent ALC rise: tHL group (transient hyperlymphocytosis) had short-lived lymphocytosis (ALC M3 *<* ALC M0) and pHL group displayed a prolonged hyperlymphocytosis (ALC M3 *>* ALC M0) (Fig 1B). This 3-month cut-off was chosen in agreement with the RESONATE and RESONATE-2 trials, where median time to recovery from pHL was 14 and 12 weeks, respectively. Out of 122 patients treated by ibrutinib 68% were in tHL group versus 32% in pHL group in cohort 1 (*n* = 41) and 57% in tHL group versus 43% in pHL group in cohort 2 (*n* = 81) (Chi^2^: 0.144) (Fig 1C). Factors significantly associated with pHL were: lower median pre-treatment ALC, absence of deletion 17p, presence of deletion 11q, and older median age but not immunoglobulin heavy chain variable region genes mutational status (S1 Fig).

Differential lymphocytosis in our series was not associated with progression-free survival (PFS) or overall survival (OS). So, we investigated patients’ disposition along time of treatment exposition by swimmer plot analyses (S2 Fig). Furthermore, before 1 year of treatment, aggressive disease (Richter syndromes) occurred more frequently in tHL group (9%) than in pHL group (0%), whereas percent of CLL progression was not clearly different in both groups (6% in tHL group versus 4% in pHL group) (S4 Table). Grade 5 (lethal) adverse events occurred also more frequently in tHL group before 1 year of treatment (7% versus 0%, respectively). In addition, before 6 months, infections (Grade ≥ 3) were more frequent in tHL group (37%) than in pHL group (24%) (*p* = 0.04) and in relapsed/refractory patients than first-line (34% versus 15%, respectively; *p* = 0.003) (S4 Table). No correlation was found between infections and levels of B leukemic cells, CD4^+^ and CD8^+^ T lymphocytes, NK cells and monocytes or other clinico-biological parameters.

Interestingly, we could calculate in 91 patients (tHL group *n* = 49, pHL group *n* = 42) the BALL score [33], that is related to 4 associated factors (1 point each): ß2-microglobulin (>5 mg/L), Anemia (Hemoglobin <110 g/L), Lactacte Deshydrogenase (LDH > upper limit of normal), Last therapy (time from initiation of last therapy < 24 months). This score has been described to predict OS under ibrutinib (irrespectively of del(11q)/del(17p) status) in relapsed/refractory CLL registration trials [34]. In our post hoc analysis, we observed that distribution of low (40% versus 60%), intermediate (59% versus 41%), and high risk (80% versus 20%) scores was statistically different between the groups (Fig 1D). Our data show that patients with pHL were overrepresented in the BALL low-risk group (60% versus 40%) and underrepresented in the high-risk group (20% versus 80%) compared with patients with tHL, suggesting that pHL group might have a better prognosis at the population level.

### Baseline immune contexture and PD1 levels relate to pHL group

**Fig 2:**
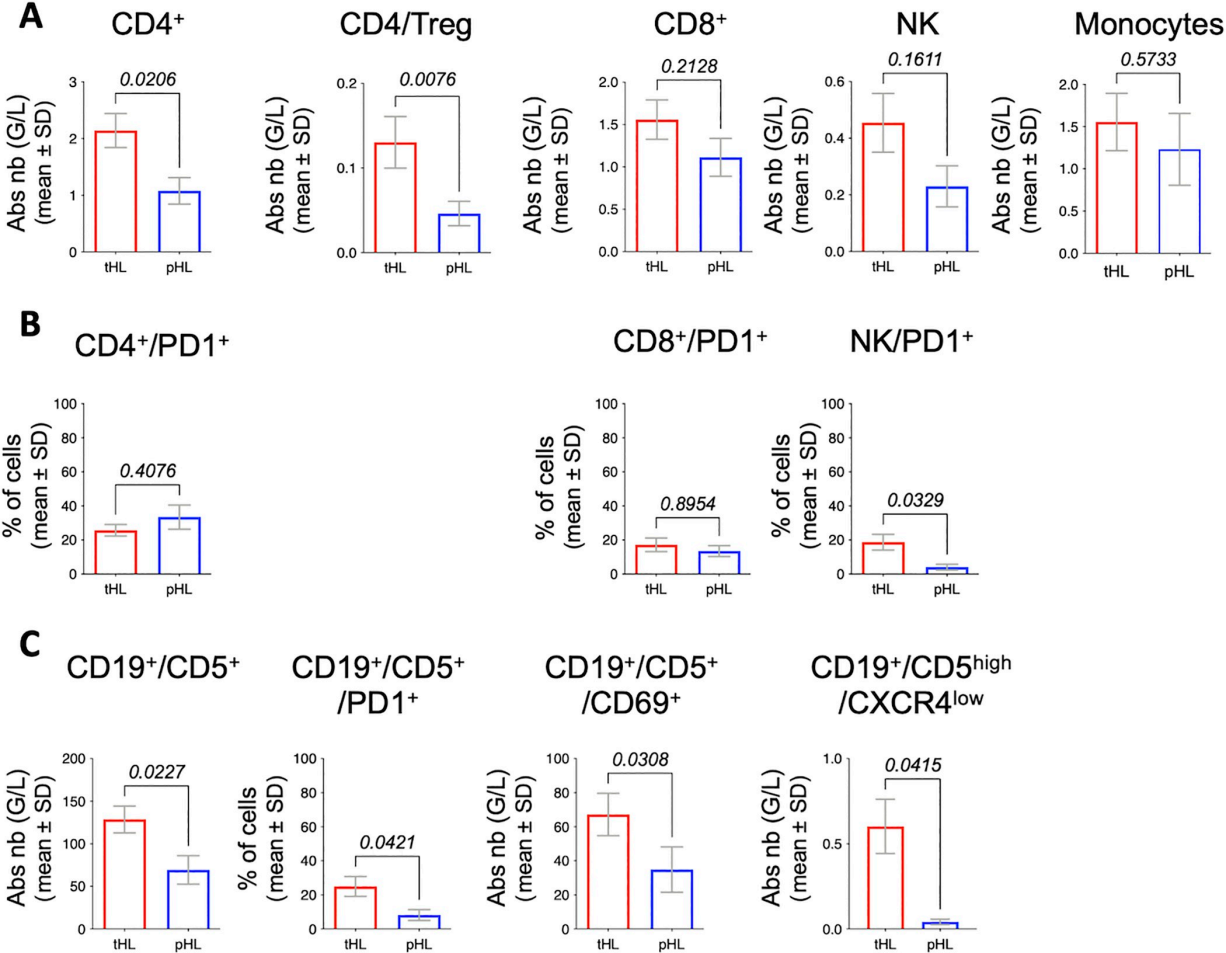
Immuno-phenotyping of CLL populations before ibrutinib treatment according to tHL and pHL groups. **(A)** Absolute number of CD4 lymphocytes, regulatory CD4 T cells (CD4/Treg), CD8 lymphocytes, Natural Killer cells (NK), and monocytes; G/L: Giga per Liter. (B) Percentage of immune cells expressing PD1. (C) Immuno-phenotyping of B leukemic cells. Abs nb, Absolute number; G/L, Giga per Liter; SD, standard deviation. For all graphs: two-tailed Mann–Whitney test between tHL group (red) and pHL group (blue). CLL, chronic lymphocytic leukemia; pHL, prolonged hyperlymphocytosis; tHL, transient hyperlymphocytosis.

Monitoring of normal immune cells subsets was done in the blood of patients from cohort 1 and revealed significant differences in some cellular populations according to the groups. We could further show that baseline absolute CD4^+^ T lymphocytes and regulatory CD4 T (Tregs) cell counts were associated with pHL (Fig 2A), but Tregs cell counts were not correlated with CD4^+^ T lymphocytes absolute number (tHL group R^2^ = 0.009; pHL group R^2^ = 0.05). Furthermore, baseline PD1 level, at the surface of B leukemic lymphocytes and NK cells, was also associated with pHL (Fig 2B and 2C). As already reported [6], baseline CD19^+^/CD5^+^ level was correlated with pHL (Fig 2C). pHL group exhibited less baseline activated B leukemic cells (CD69^+^) (Fig 2C), with respect to B leukemic cells level (tHL group R^2^ = 0.591, *p* < 0.0001; pHL group R^2^ = 0.591, *p* = 0.003). Finally, pHL group displayed also lower circulating B leukemic cells exhibiting lymph node features (CD19^+^/CD5^high^/CXCR4^low^) (Fig 2C) that were not correlated with CD19^+^/CD5^+^ cell count (tHL group R^2^ = 0.17, *p* = 0.09; pHL group R^2^ = 0.17, *p* = 0.44). Baseline percentage of PD1^+^ CLL cells before treatment was also associated to risk of infection. Indeed, infections occurred more frequently in patients with PD1^+^ CLL cells < 20% (80% versus 42%, respectively; *p* < 0.0001).

In order to “explore” the lymph node compartment, MRI parameters were assessed in cohort 1 patients who accepted to participate to this exploration (*n* = 26). LN volumes did not significantly discriminate tHL and pHL groups (S3 Fig). Similarly, spleen volumes were not significantly different between both groups (*p* = 0.928). We also measured the ADC on the first 9 patients, before ibrutinib treatment. We observed that tHL group patients presented a higher ADC (related to lower cellularity) than patients from pHL group (*p* = 0.03) without correlation with ALC (R^2^ = 0.0005) (S3 Fig).

### Cell population dynamics during ibrutinib treatment

**Fig 3:**
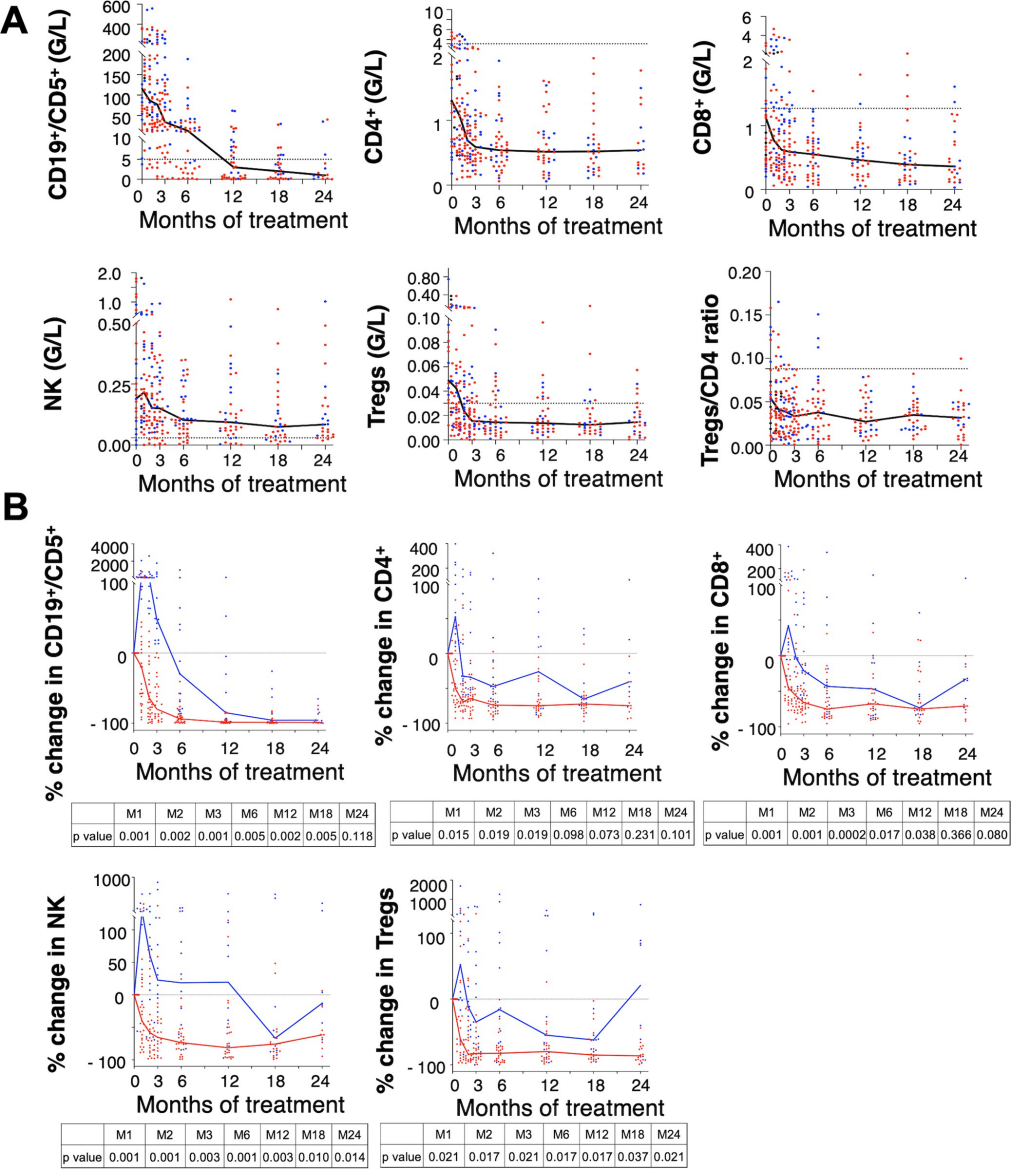
Immuno-monitoring of CLL cell populations during ibrutinib treatment. **(A)** Median (black line) of absolute number in leukemic B cells (CD19^+^/CD5^+^), CD4 lymphocytes, CD8 lymphocytes, Natural Killer (NK) cells, regulatory CD4 T cells (Tregs), and Tregs/CD4 ratio. In each subgraph, dotted line represents the median of absolute number of cells in healthy donors (for T lymphocytes and NK cells) and in CLL at diagnosis (B CD19^+^/CD5^+^); G/L: Giga per Liter. Red dots (tHL group) and blue dots (pHL group) represent each patient at different time point. **(B)** Median of percent change in leukemic B cells (CD19^+^/CD5^+^), CD4 lymphocytes, CD8 lymphocytes, Natural Killer (NK) cells, regulatory CD4 T cells (Tregs) compared to baseline in tHL group (red) and pHL group (blue). G/L: Giga per Liter. Red dots (tHL group) and blue dots (pHL group) represent each patient at different time point. M1 to M24 indicate month 1 up to month 24 of treatment. Statistics: two-tailed Mann–Whitney test between tHL and pHL groups. CLL, chronic lymphocytic leukemia; pHL, prolonged hyperlymphocytosis; tHL, transient hyperlymphocytosis.

Evolution of cellular populations in blood was followed during ibrutinib treatment in cohort 1 (Fig 3A). Considering all patients, ibrutinib induced a decrease of all cellular populations along time of treatment. To note, T lymphocyte counts never went back to levels observed in healthy donors, even after long-term ibrutinib treatment reflecting the effect of interleukin-2-inducible T-cell kinase (ITK) targeting. In addition, even if NK cell counts decreased upon ibrutinib treatment, they remained elevated as compared to healthy donors (Fig 3A).

As observed for ALCs, ibrutinib treatment induced 2 profiles of response for all cellular populations according to tHL or pHL groups (Fig 3B). Indeed, all subsets increased rapidly in pHL group, probably due to cellular egress from lymph nodes. However, kinetic of cellular decrease was different between B and T lymphocyte populations showing that the hyperlymphocytosis of T cells in pHL group was more limited in intensity and duration than what is observed for B leukemic cells and NK subsets.

**Fig 4:**
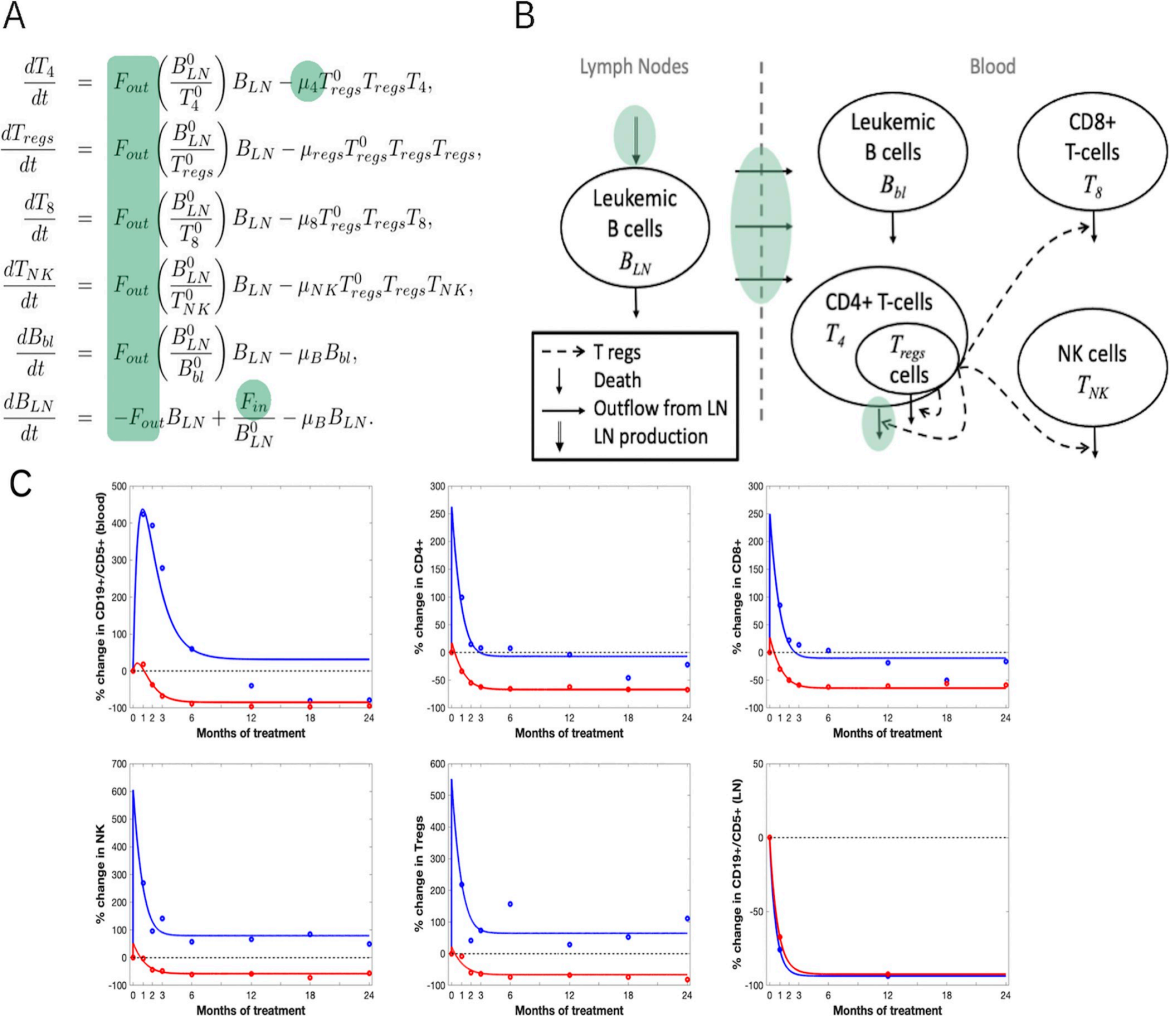
Mathematical model to simulate cell dynamics in CLL under ibrutinib treatment. **(A)** Mathematical system describing B and T cell dynamics. All variables are introduced in Table 1 and details provided in S1 File Modeling. **(B)** Schematic representation of the mathematical model. In (A) and (B), the green areas highlight the processes that depend on the groups when using the nonlinear mixed-effects model. **(C)** Both simulated (plain lines) and clinical (dots, mean values) data of B and T cell dynamics in blood are displayed, as well as B cell dynamics in lymph nodes (bottom right) in tHL group (red) and pHL group (blue). Simulated dynamics have been obtained with parameter values in S5 Table. CLL, chronic lymphocytic leukemia; LN, lymph nodes; NK, Natural Killer cells; pHL, prolonged hyperlymphocytosis; tHL, transient hyperlymphocytosis; Tregs, regulatory CD4 T cells.

We built a mathematical model of B and T cell dynamics from cohort 1 clinical measurements. The model has been developed in several steps, by incorporating biological and medical knowledge on CLL and its treatment based on ibrutinib. Important, and sometimes prolonged, increase of B or T cell counts in blood following the onset of the treatment is observed in the cohort. Ibrutinib is known to deplete lymph nodes of leukemic B cells, so we hypothesized that not only B but also T cells exit the lymph nodes following ibrutinib treatment. Thanks to available information, we assumed that for each T cell population the flux of cells from the lymph nodes is proportional to the number of leukemic B cells in the lymph nodes (*B*_*LN*_). This led to equations describing B and T cell dynamics, where all cell counts are normalized by their initial value (Fig 4A). A schematic representation of the model is presented in Fig 4B.

The average behavior of patients from each group is correctly reproduced (Fig 4C). Even though in both cases leukemic B cell counts in blood are overestimated from M6 after the onset of the treatment, specific dynamics of both groups are well described. The pHL characterizing pHL group is clearly visible, and is associated with high T cell counts up to M1/M2. Dynamics of tHL group show a very brief hyperlymphocytosis (less than 1 month), associated with an increase of T cell counts on the same period. This result cannot be compared to clinical measurement yet due to lack of measurements between M0 and M1.

Parameter values estimated to fit patient’s data and generate cell dynamics in Fig 4C are listed in S5 Table. The estimated values show first that T cell renewal rates are similar in both groups, suggesting no group-specific influence of ibrutinib on T cell renewal. Second, they highlight that the main difference between both groups is in the exit rate of leukemic B cells from lymph nodes: it is 8 times larger in pHL group. Also, patients from pHL group have lower B cell death and production rates than patients from tHL group.

Based on the mathematical model built for describing average clinical measurements, we can assess that T cell count dynamics in blood are impacted by lymph node cell counts dynamics. In addition, in blood, no interaction between leukemic B cells and T cells was required to explain the observed dynamics, suggesting that these interactions can be neglected.

### Biological parameters under ibrutinib treatment

**Fig 5:**
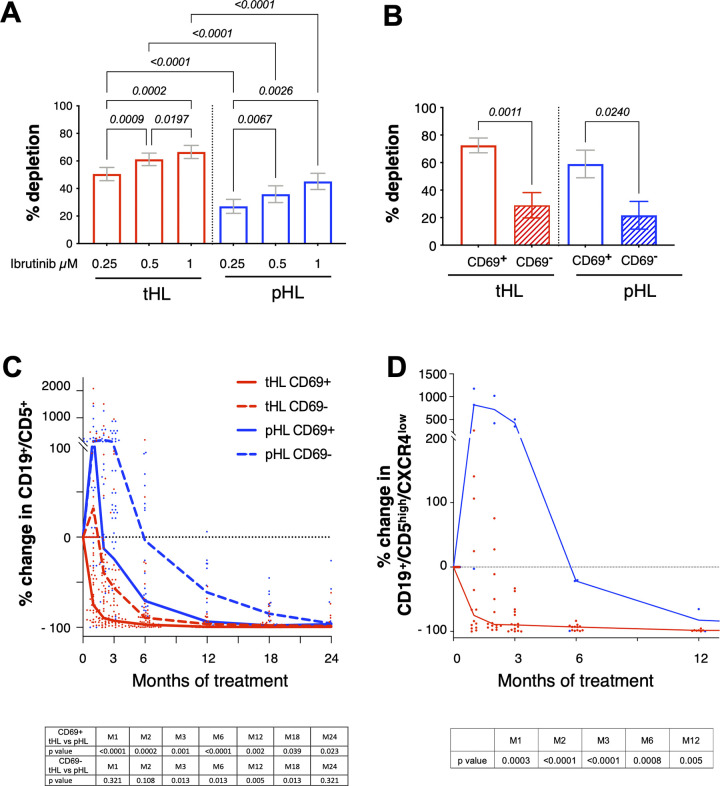
In vitro ibrutinib sensitivity and egress of B leukemic cells. **(A)** In vitro dose-effect of ibrutinib on B leukemic cell depletion at M0. Statistics: two-tailed Mann–Whitney test; SD, standard deviation. **(B)** In vitro ibrutinib-sensitivity of B leukemic cells according to CD69 expression at M0. Statistics: two-tailed paired Student *t* test; SD, standard deviation. **(C)** Median (tHL group, red; pHL group, blue) of percent change of B leukemic cells, according to CD69 expression, under ibrutinib exposure. Red dots (tHL group) and blue dots (pHL group) represent each patient at different time points. Statistics: two-tailed Mann–Whitney test between tHL and pHL groups. **(D)** Median (tHL group, red; pHL group, blue) of percent change of lymph node circulating B leukemic cells during ibrutinib treatment. Red dots (tHL group) and blue dots (pHL group) represent each patient at different time points. Statistics: two-tailed Mann–Whitney test between tHL and pHL groups. pHL, prolonged hyperlymphocytosis; tHL, transient hyperlymphocytosis.

In addition to a better egress of lymphoid cells in patients from pHL group, it could be also possible that B leukemic cells from these patients are less sensitive to ibrutinib due to a differential expression of BTK or its phosphorylated form. Before treatment, no difference was observed in BTK expression and pBTK/BTK ratio between both groups. In both groups, 1-month ibrutinib exposure led to a decrease of BTK protein level associated with an increase of its phosphorylated form, followed by a decrease of pBTK/BTK ratio after M6 (S4 Fig). Before treatment, in vitro depletion assays confirmed that ibrutinib induced less cell death in pHL group than in tHL group, irrespective of ibrutinib concentration (Fig 5A). In addition, ibrutinib is more efficient to CD69^+^ leukemic cells in both groups (Fig 5B). Accordingly, monitoring of CD69^+^ and CD69^*−*^ showed a significantly sustained level of CD69^*−*^ B leukemic cells in pHL group along ibrutinib exposure (Fig 5C). In addition, pHL was associated with a higher egress of B leukemic cells from lymph nodes to blood as observed by the rate of CD5^high^CXCR4^low^ circulating cells (Fig 5D), with a significant increase of CD5^high^CXCR4^low^CD69^−^ during the first 6 months of treatment (M1 *p* = 0.0003; M2 *p* < 0.0001; M3 *p* < 0.0001; M6 *p* = 0.0008). After 2 years, all patients remaining under ibrutinib treatment decreased their lymphocytosis compared to baseline level despite some patients (27%) exhibited an ALC *>* 5 G/L (Fig 1A).

### Mathematical modeling highlights 3 biological processes specific to pHL group

Our cohort resulted in patient-specific data for cohort 1 patients, consisting in cell counts for various cell types (B and T cells in blood and estimated B cell counts in lymph nodes) at different times after the onset of the treatment. Such data exhibit important inter-patient variability (Fig 1A). To account for it, we introduced a population approach with NLME modeling [26,30]. Contrary to the model on average data, here each patient dynamics is taken into account.

We found that the NLME model that best reproduces the measurements requires patient-specific parameters for all B and T cell processes and includes the group covariate. All T cell mortality parameters are correlated (*μ*_4_, *μ*_8_, *μ*_*NK*_, *μ*_*reg*_), as well as production and outflow of the leukemic B cells in lymph nodes (*F*_*in*_, *F*_*out*_). S6 Table summarizes estimated parameters.

Noticeably, *F*_*in*_, *F*_*out*_ and *μ*_4_ are group specific (Fig 4B), highlighting that patients from tHL and pHL groups differ significantly in their leukemic B cell dynamics within lymph nodes. Patients from pHL group are characterized by a much higher efflux rate from lymph nodes (44 times higher than in tHL group). The lymph node production rate is 5 times lower in pHL group than tHL group. Finally, CD4^+^ T cell mortality rates also differ between groups, yet differences are less important (death rate 1.3 times higher in pHL group). The absence of correlation between parameters governing B and T cell dynamics shows that these dynamics are independent in blood. In addition, the death rate of leukemic B cells is the same in both groups showing that differences between both groups do not rely on this process.

**Fig 6:**
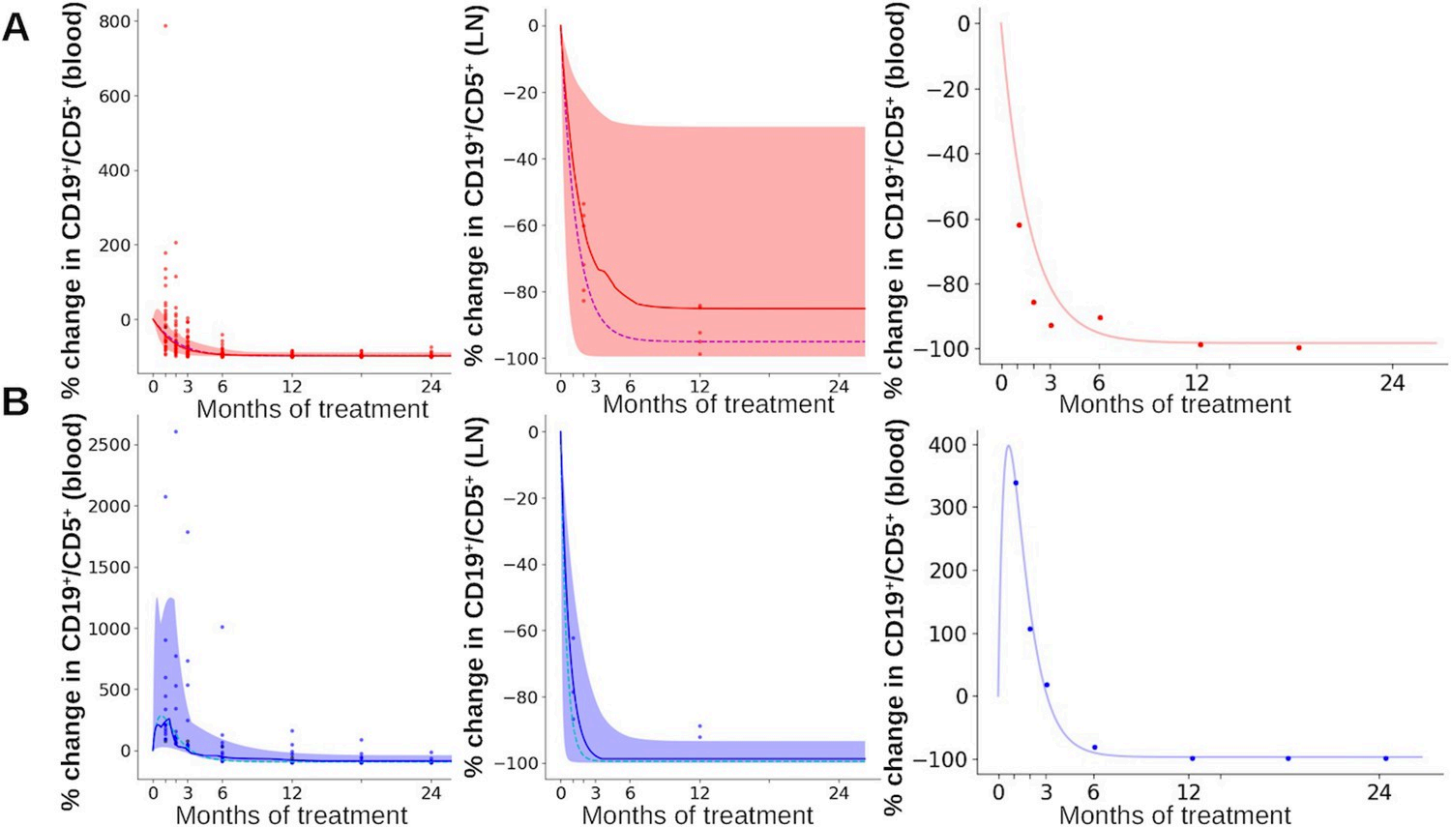
Model predictions of B leukemic cell evolution accounting for inter-patient variability. **(A)** Percent change in CD19^+^/CD5^+^ cell counts, in blood (left) and LNs (center), and an example of an individual specific dynamic displayed for CD19^+^/CD5^+^ cell counts in blood (right) in tHL group. **(B)** Percent change in CD19^+^/CD5^+^ cell counts, in blood (left) and LNs (center), and an example of an individual specific dynamic displayed for CD19^+^/CD5^+^ cell counts in blood (right) in pHL group. Dynamics are obtained from simulations of the nonlinear mixed-effects model (Fig 4A and 4B), using parameter values from S6 Table. In each plot, individual clinical measurements appear as dots. Median and mean cell counts (left, center) are represented by a straight and dashed line respectively, 10th and 90th percentiles by the colored shaded area, computed from model predictions. LN, lymph node; pHL, prolonged hyperlymphocytosis; tHL, transient hyperlymphocytosis.

Fig 6 displays measured leukemic B cell counts in blood and lymph nodes for patients from tHL and pHL groups and the average cell count computed from the model. Also, individual dynamics predicted by the model were used to compute the median, the 90th and 10th percentiles. An example, for each group, of individual leukemic B cell counts in blood is displayed. It shows the ability of the NLME model to reproduce patient-specific dynamics. It highlights the good quality of model prediction when patient variability is accounted for. Results in lymph nodes, for both groups, are impacted by the limited number of measurements. Quality of model predictions is similar for both groups.

## Discussion

Our report illustrates that interdisciplinarity between biology, medical imaging, and mathematics is a strength to better understand and explain clinical observations. Indeed, in our longitudinal study and in accordance to previous clinical observations [8,9], we confirmed that ibrutinib treatment in CLL patients induced 2 patterns of response: a transient and a prolonged lymphocytosis. We highlighted the importance of tumor microenvironment in the 2 types of response and the disease course upon ibrutinib treatment for the 2 patterns of response. Indeed, we identified clinical baseline parameters (age and absolute lymphocyte counts), biological features such as absolute counts of normal immune cells (CD4^+^ T lymphocytes, Treg cells), CD19/CD5^high^/CXCR4^low^ B leukemic cells, PD1 and CD69 expression on B leukemic hyperlymphocytosis and lymph node cellularity assessing by whole body MRI. We also pinpointed that the group of patients identified by the transient hyperlymphocytosis had lower duration response and a poor clinical outcome. By developing the mathematical model, we confirmed the role of the balance between cell death and cell egress from lymph nodes in the response to ibrutinib treatment. Noticeably, this study was not based on statistical dichotomization of patients but built on previous clinical reports, defining the 2 groups of response under ibrutinib treatment. Nevertheless, the model confirmed cell counts evolution of B leukemic cells [5,7] and provided new information on T lymphocytes dynamics in CLL patients upon ibrutinib treatment. Thus, our study gave a better knowledge of ibrutinib response in CLL patients and provided information for clinicians to better manage ibrutinib therapy, especially in the first 3 months of treatment.

Our study relied on ALCs-based groups of response to treatment, with groups either exhibiting pHL (ALC M3 > ALC M0) or tHL (ALC M3 < ALC M0). Similar definition of groups of patients was previously introduced in clinical studies [6,8–10]. In this work as well as in previous ones [6,8–10], dichotomization of patients in 2 groups has not been performed based on a statistical criterion. Indeed, statistical methods could be used to determine whether 2 or more groups of patients can be extracted from the data, and if so, what would be the optimal criterion to define the groups. For instance, mixture models with different numbers of components could be fit to the data [35,36]. Since distributions of ALCs are highly skewed to the right (few patients with very high ALCs), it is however important to account for this specific feature of the data that make them unlikely to be fitted by Gaussian distributions. We nevertheless decided to use group definitions introduced in the literature, since they represented clinical practice and consequently had potential easy clinical applications by helping to better stratify and follow patients during ibrutinib treatment. In addition, it may be noted that ALC data were not normally distributed (Shapiro–Wilk test, not shown) and did not have the same laws (Wilcoxon Mann–Whitney test, not shown) at M0, M2, and M3, suggesting that the 2 groups identified different treatment responses.

Since this study was based on only 1 measurement per time point and per patient, it was therefore not possible to estimate the regression to the mean effect [37] in measured ALCs and groups definition. However, over the 122 patients of both cohorts 1 and 2, dynamics over the 3 first months after treatment (measurements M1, M2, and M3) remained the same for 90 patients (that is, either above or below baseline). We could then assume that the variations on which groups of responses had been constructed were mostly due to the treatment effect and could not be explained by regression to the mean effect only.

Introducing a mathematical model to describe cell counts evolution in CLL patients treated with ibrutinib was an original and complementary approach to the current clinical study. We chose to introduce an NLME model to characterize each patient profile instead of independently fitting our model to individual patient data. Indeed, such an approach would have been limited by missing data that could prevent model parameters identifiability. The NLME approach used efficiently the available information from existing measurements. Estimated values for leukemic B cell death rate and the exit rate from lymph nodes were of the same order of magnitude than the ones reported in Wodarz and colleagues [5] and Burger and colleagues [7]. We additionally described T lymphocytes dynamics under ibrutinib hence characterizing more specifically the effect of ibrutinib treatment on CLL patients. We found that T cell mortality parameters are correlated, with higher correlation rates for CD4^+^/CD8^+^ T cells and regulatory CD4 T cells. To confirm its robustness, the model should be compared to a new and larger dataset.

By targeting ITK, ibrutinib also induced 2 groups of response in normal cells. This suggested that ibrutinib sensitivity and/or activation/exhaustion status of normal cells may affect the dynamic of efflux versus production rates as well as cell death. Indeed, the observed baseline of absolute CD4^+^ T cell counts was significantly smaller in pHL group. It was estimated, with the mathematical model, that the death rate of CD4^+^ T cells was higher in pHL group, contributing to explain the differences observed between groups. For each group, neither basal nor time-course level of immune cells were correlated to ALCs and, except for Natural Killer cells, absolute number of immune cells was always lower than in healthy donors. Even though T lymphocytes function was under the scope of our study, results from clinical trials using CD19/CD3 bispecific antibody [38] showed that immune cells were efficient against ibrutinib-resistant B leukemic cells. This suggested that despite a drastic reduction of T lymphocytes number by ibrutinib treatment, function was not completely altered. It would be interesting to follow immune cells time-course under new BTK inhibitors (acalabrutinib [39], zanubrutinib [40]), which induced a lesser inhibition of ITK.

In order to explain the differential response to ibrutinib, we addressed the hypothesis that lymph node micro-environment composition and related-cellularity, measured by the ADC, could be different between groups. Our results showed that patients from tHL group present a higher ADC (related to lower cellularity) than patients from pHL group without correlation with ALCs. These preliminary data should be validated in a larger cohort of patients to characterize ADC as a predictive marker of ibrutinib response evolution and its use in clinical practice. However, we can speculate that in tHL group, the structure of lymph nodes may be less compact, promoting, at least in part, the exit of cells into blood compartment before treatment. Conversely, a lower ADC (related to higher cellularity) in pHL group could be linked to a more compact structure of lymph nodes in these patients and so, a basal lower egress of cells into blood. This hypothesis is reinforced by absolute lymphocyte quantification and the high number of circulating CD19/CD5^high^/CXCR4^low^ cells in tHL group versus pHL group before treatment. Then, during ibrutinib treatment, a lower compaction of lymph nodes could promote a better diffusion of ibrutinib into these organs, leading to an increase of cell death in situ, and a reduced egress of leukemic cells (as observed in tHL group). On the contrary, a higher compaction of lymph nodes (pHL group) could decrease ibrutinib diffusion and efficiency, leading to a more prolonged egress of cells from lymph nodes to the blood compartment. However, this phenomenon could also be coupled to chemokine sensitivity and surface proteins, such as CD69, S1P1, CCR7 [41–45] that contribute to cellular egress or retention in CLL lymph nodes. Finally, the pHL during treatment could also be linked to the lesser sensitivity of CD69^-^ B leukemic cells to ibrutinib.

Mathematical results supported the biological parameters associated with pHL and the previous hypotheses. Under ibrutinib treatment, the lymph node exit rate of leukemic B cells was much higher in pHL group than in tHL group. In addition, the observed smaller leukemic B cell number in pHL group (less baseline activated leukemic B cells CD69^+^ and circulating leukemic B cells with lymph node features (CD19^+^/CD5^high^/CXCR4^low^)) could be explained by a smaller production rate in lymph nodes that was mathematically estimated to a lower value in pHL group than in tHL group. The mathematical analysis also highlighted that inter-patient variability was not sufficient to explain the differences between the 2 groups. This means that differences observed in measurements were mainly due to a significant difference in the biological processes between the 2 groups of patients.

Our study also pinpointed that blood lymphocytosis induced by ibrutinib is associated to duration response and outcomes. Indeed, pHL identified a group with a better prognosis (Fig 1) [8,10]. This observation could be related to the concept of inter-clonal equilibrium/competition among B leukemic clones leading to the non-expansion of a dominant clone (BTK mutated or more proliferating) [46–48].

Altogether, our study provides a better understanding of which baseline clinico-biological and pharmaco-dynamic parameters dictate absolute lymphocyte kinetics upon ibrutinib treatment. Combining MRI and standard procedures (such as minimal residual disease in blood and bone marrow compartments) might help to design future clinical trials with a more thorough evaluation of “tissular compartments” responses. As our results highlight suboptimal outcomes in patients with transient hyperlymphocytosis, we believe that our approach could provide a support for choosing whether ibrutinib should be given alone or associated with, for instance, venetoclax (this combo is approved by EMEA, not yet FDA) or other therapies after 3 months.

## Supporting information

S1 STROBE Checklist STROBE Statement—checklist of items that should be included in reports of observational studies. (PDF)

S1 Table Antibodies used in the study. (PDF)

S2 Table Initial configurations of nonlinear mixed-effects (nlme) models used with SAMBA. The second column indicates which error model has been used (Constant or Proportional). The third column indicates which random effects (r.e) were accounted for. The fourth column indicates which correlations were considered. The fifth column indicates whether covariates were included, and if so on which parameters. Parameters are described in Table 1 (main text).
(PDF)

S3 Table Clinical characteristics of patients. tHL, transient hyperlymphocytosis group; pHL, prolonged hyperlymphocytosis group. Nb, number; Tt, treatment; LN, lymph nodes.
(PDF)

S4 Table Clinical outcome of CLL patients according to transient hyperlymphocytosis (tHL) and prolonged hyperlymphocytosis (pHL) groups. Nb, number.
(PDF)

S5 Table Parameter values associated with the best fit of average clinical data for both transient hyperlymphocytosis (tHL) and prolonged hyperlymphocytosis (pHL) groups and used in Fig 4C. Parameters are described in Table 1 (main text). (CI—confidence intervals, based on the profile likelihood; d–day).
(PDF)

S6 Table Parameter values associated with the best fit of individual patient measurements. Values are averaged over 10 runs with different initial guess. Parameters are described in Table 1 (main text) and in S1 File Modeling. For fixed parameters F_*out*_, F_*in*_ and *μ*_*4*_, superscripts *t* and *p* refer to tHL (transient hyperlymphocytosis) and pHL (prolonged hyperlymphocytosis) groups, respectively, identified by a covariate (see Section 2.2 in S1 File Modeling). Coefficient *c*(*x*, *y*) is the correlation coefficient between *x* and *y* computed by Monolix (see section 2.1 in S1 File Modeling). (SD–standard deviation; LL–log-likelihood; d–day; NU—no unit).
(PDF)

S1 Fig Analysis of factors associated with hyperlymphocytosis. Median of percent change in age **(A)** and absolute lymphocyte counts (ALCs) **(B)** (insert: according to cohort 1 and 2) in transient hyperlymphocytosis (tHL) and prolonged hyperlymphocytosis (pHL) groups; each dot represents a patient. **(C)** Percent of patients in tHL (red) and pHL (blue) groups according to genetic alterations. Del: deletion; IGHV M: mutated immunoglobulin heavy chain variable region genes; IGHV UM: unmutated immunoglobulin heavy chain variable region genes.
(PDF)

S2 Fig Long-term evolution of patients under ibrutinib therapy. Transient hyperlymphocytosis group (tHL) (*n* = 68); prolonged hyperlymphocytosis group (pHL) (*n* = 52); each line represents a patient.
(PDF)

S3 Fig Magnetic resonance imaging (MRI) analysis before ibrutinib treatment. **(A)** Representative features of total body MRI for a patient before ibrutinib treatment. Arbitrary colors indicate the different organs of interest (cervical, axillary, mediastinal, retroperitoneal, iliac lymph nodes, spleen, and liver). **(B)** Volume of lymph nodes according to transient hyperlymphocytosis group (tHL) and prolonged hyperlymphocytosis group (pHL). **(C)** ADC of lymph nodes according to tHL and pHL groups. SD, standard deviation.
(PDF)

S4 Fig Monitoring of phosphoBTK/BTK along ibrutinib treatment. tHL, transient hyperlymphocytosis group; pHL, prolonged hyperlymphocytosis group; SD, standard deviation; a.u., arbitrary units.
(PDF)

S1 File Modeling Model construction, parameter estimation, model selection. Details on modeling choices, parameter estimation methodology, model selection procedure, for both the generic model and the patient-specific model.
(PDF)

## Abbreviations

ADC: apparent diffusion coefficient
ALC: absolute lymphocyte count
CLL: chronic lymphocytic leukemia
ITK: interleukin-2-inducible T-cell kinase
LN: lymph node; MRI, magnetic resonance imaging
NLME: nonlinear mixed effect
ODE: ordinary differential equation
OS: overall survival
PBMC: peripheral blood mononuclear cell
PFS: progression-free survival
pHL: prolonged hyperlymphocytosis
SAMBA: Stochastic Approximation for Model Building Algorithm
tHL: transient hyperlymphocytosis
