# Supplementary material for: New pharmacodynamic parameters linked with ibrutinib responses in chronic lymphocytic leukemia: Prospective study in real-world patients and mathematical modeling
Source: PLoS Med. 2024 Jul 22;21(7):e1004430. doi: 10.1371/journal.pmed.1004430 (PMC11262688; doi:10.1371/journal.pmed.1004430)
Supplement: S1 Table — (PDF) [file pmed.1004430.s002.pdf]

Company	Antibody	Clone
BioLegend	Brilliant Violet 421 anti-human CD19	HIB19
	PE-Cy7 anti-human CD5	UCHT2
	Pacific Blue anti-human CD3	OKT3
	PE anti-human CD8	SK1
	PE anti-human CD69	FN50
	APC anti-human CD184	12G5
	Brilliant Violet 421 Mouse IgG1 isotype control	MOPC-21
	PE-Cy7 Mouse IgG1 isotype control	MOPC-21
	Pacific Blue Mouse IgG1 isotype control	MOPC-21
	PE Mouse IgG1 isotype control	MOPC-21
	APC Mouse IgG2a isotype control	MOPC-173
BD Biosciences	PE-Cy5 anti-human CD4	RPA-T4
	PE-Cy7 anti-human CD56	B159
	FITC anti-human CD279	MIH4
	PE-Cy7 anti-human CD25	M-A251
	FITC anti-human CD127	HIL-7R-M21
	Phosflow anti-BTK (pY223) / ITK(pY180)	N35-86
	AlexaFluor 647 anti-human BTK	53/BTK
	PE-Cy5 Mouse IgG1 isotype control	MOPC-21
	PE-Cy7 Mouse IgG1 isotype control	MOPC-21
	FITC Mouse IgG1 isotype control	MOPC-21
	PE Mouse IgG1 isotype control	MOPC-21
	AlexaFluor 647 Mouse IgG1 isotype control	MOPC-21

S1 Table: **Antibodies used in the study**
