# Supplementary material for: New pharmacodynamic parameters linked with ibrutinib responses in chronic lymphocytic leukemia: Prospective study in real-world patients and mathematical modeling
Source: PLoS Med. 2024 Jul 22;21(7):e1004430. doi: 10.1371/journal.pmed.1004430 (PMC11262688; doi:10.1371/journal.pmed.1004430)
Supplement: S2 Table — The second column indicates which error model has been used (Constant or Proportional). The third column indicates which random effects (r.e) were accounted for. The fourth column indicates which correlations were considered. The fifth column indicates whether covariates were included, and if so on which parameters. Parameters are described in Table 1 (main text). (PDF) [file pmed.1004430.s003.pdf]

Initial nlme model	Error model	Random effects (r.e)	Correlations of r.e	Covariates
$nlme_1$	Constant	None	None	None
$nlme_2$	Constant	All	None	All parameters
$nlme_3$	Constant	μ_B, μ_4, μ_8, μ_{NK}, μ_{reg}	None	None
$nlme_4$	Constant	F_{out}, F_{in}	None	F_{in}, μ_B
$nlme_5$	B_{LN}: Constant<br>B_{bl}, T: Proportional	All	(F_{out}, F_{in})	F_{out}, F_{in}, μ_4

S2 Table: **Initial configurations of nonlinear mixed-effect (nlme) model used with SAMBA [1].** The second column indicates which error model has been used (Constant or Proportional). The third column indicates which random effects (r.e) were accounted for. The fourth column indicates which correlations of random effects were considered. The fifth column indicates whether covariates were included, and if so on which parameters. Parameters are described in Table 1 (main text).

Reference:

[1] Prague M, Lavielle M. SAMBA: A novel method for fast automatic model building in nonlinear mixed-effects models. CPT Pharmacom & Syst Pharma. 2022 Feb;11(2):161–72.
