# Supplementary material for: New pharmacodynamic parameters linked with ibrutinib responses in chronic lymphocytic leukemia: Prospective study in real-world patients and mathematical modeling
Source: PLoS Med. 2024 Jul 22;21(7):e1004430. doi: 10.1371/journal.pmed.1004430 (PMC11262688; doi:10.1371/journal.pmed.1004430)
Supplement: S3 Table — tHL, transient hyperlymphocytosis group; pHL, prolonged hyperlymphocytosis group. Nb, number; Tt, treatment; LN, lymph nodes. (PDF) [file pmed.1004430.s004.pdf]

	Cohort 1	Cohort 2	Total Cohort (1 + 2)	Total Cohort tHL group	Total Cohort pHL group
Number of patients	*41*	*81*	*122*	*68*	*54*
	Nb (%)	Nb (%)	Nb (%)	Nb (%)	Nb (%)
Sex M	27 (66%)	55 (66%)	82 (68%)	49 (72%)	33 (61%)
F	14 (34%)	26 (32%)	40 (33%)	19 (28%)	21 (39%)
Number of patients	*41*	*81*	*122*	*68*	*54*
Median age (yrs)	70	70	70	68	73
Number of patients	*41*	*81*	*122*	*68*	*54*
	Nb (%)	Nb (%)	Nb (%)	Nb (%)	Nb (%)
First Line	14 (34%)	20 (25%)	33 (27%)	21 (31%)	12 (22%)
Relapsed/Refractory	27 (66%)	61 (75%)	89 (73%)	47 (69%)	42 (78%)
Number of patients	*41*	*73*	*114*	*65*	*49*
	Nb (%)	Nb (%)	Nb (%)	Nb (%)	Nb (%)
IGHV mutational status UM	31 (76%)	56 (77%)	87 (76%)	52 (80%)	35 (71%)
M	10 (24%)	17 (23%)	27 (24%)	13 (20%)	14 (29%)
Number of patients	*40*	*75*	*115*	*66*	*49*
	Nb (%)	Nb (%)	Nb (%)	Nb (%)	Nb (%)
Del 17p	20 (50%)	26 (35%)	46 (40%)	32 (48%)	14 (29%)
Number of patients	*40*	*75*	*115*	*66*	*49*
	Nb (%)	Nb (%)	Nb (%)	Nb (%)	Nb (%)
Del 11q	15 (38%)	31 (41%)	46 (40%)	22 (33%)	24 (49%)
Number of patients	*41*	*69*	*110*	*62*	*48*
	Nb (%)	Nb (%)	Nb (%)	Nb (%)	Nb (%)
Complex karyotype	19 (46%)	26 (38%)	45 (41%)	26 (45%)	19 (40%)
Number of patients	*40*	*40*	*80*	*53*	*27*
	Nb (%)	Nb (%)	Nb (%)	Nb (%)	Nb (%)
Bulky (LN > 5cm)	5 (35%)	24 (60%)	38 (48%)	24 (45%)	14 (52%)
Number of patients	*40*	*38*	*78*	*51*	*27*
	Nb (%)	Nb (%)	Nb (%)	Nb (%)	Nb (%)
Bulky spleen	10 (25%)	18 (47%)	28 (36%)	20 (39%)	8 (30%)

S3 Table: **Clinical characteristics of patients.**
tHL: transient hyperlymphocytosis group; pHL: prolonged hyperlymphocytosis group
Nb: number; Tt: treatment; LN: Lymph Nodes
