# Supplementary material for: New pharmacodynamic parameters linked with ibrutinib responses in chronic lymphocytic leukemia: Prospective study in real-world patients and mathematical modeling
Source: PLoS Med. 2024 Jul 22;21(7):e1004430. doi: 10.1371/journal.pmed.1004430 (PMC11262688; doi:10.1371/journal.pmed.1004430)
Supplement: S4 Table — Nb, number. (PDF) [file pmed.1004430.s005.pdf]

	tHL group	pHL group
Number of patients	68	54
Richter Transformation	Nb (%)	Nb (%)
All cases	**11 (16%)**	**2 (3%)**
< 1 yr of treatment	**6 (9%)**	**0 (0%)**
Progressive disease	Nb (%)	Nb (%)
All cases	17 (25%)	11 (20%)
< 1 yr of treatment	4 (6%)	2 (4%)
Total Death	Nb (%)	Nb (%)
All cases	30 (44%)	26 (48%)
< 1 yr of treatment	**10 (14.7%)**	**3 (5.5%)**
Toxicity	Nb (%)	Nb (%)
All cases	18 (26%)	12 (22%)
Lethal toxicity	9 (13%)	8 (15%)
< 1 yr of treatment	**9 (13%)**	**3 (5.5%)**
Lethal toxicity	**5 (7%)**	**0 (0%)**

S4 Table: **Clinical outcome of CLL patients according to transient hyperlymphocytosis (tHL) and prolonged hyperlymphocytosis (pHL) groups.**
Nb: number
