# Supplementary material for: New pharmacodynamic parameters linked with ibrutinib responses in chronic lymphocytic leukemia: Prospective study in real-world patients and mathematical modeling
Source: PLoS Med. 2024 Jul 22;21(7):e1004430. doi: 10.1371/journal.pmed.1004430 (PMC11262688; doi:10.1371/journal.pmed.1004430)
Supplement: S5 Table — Parameters are described in Table 1 (main text). (CI—confidence intervals, based on the profile likelihood; d–day). (PDF) [file pmed.1004430.s006.pdf]

Parameter	tHL group		pHL group		Units
	value	CI	value	CI	
F_{out}	0.0041	[0.0034;0.0048]	0.031	[0.028;0.033]	d^{-1}
F_{in}	7.76	[7.75;7.77]	3.26	[3.256;3.261]	cells.d^{-1}
μ_B	0.037	[0.036;0.038]	0.021	[0.018;0.024]	d^{-1}
μ_4	21	[17;27]	23	[18;29]	$cells^{-1}.d^{-1}$
μ_8	27	[21;33]	23	[18;29]	$cells^{-1}.d^{-1}$
μ_{NK}	70	[56;87]	56	[47;67]	$cells^{-1}.d^{-1}$
μ_{reg}	351	[267;466]	270	[215;345]	$cells^{-1}.d^{-1}$

S5 Table: **Parameter values associated with the best fit of average clinical data for both transient hyperlymphocytosis (tHL) and prolonged hyperlymphocytosis (pHL) groups and used in Figure 4C.** Parameters are described in Table 1 (main text). (CI - confidence intervals, based on the profile likelihood [1]; d – day).

Reference:

[1] Raue A, Kreutz C, Maiwald T, Bachmann J, Schilling M, Klingmüller U, et al. Structural and practical identifiability analysis of partially observed dynamical models by exploiting the profile likelihood. Bioinformatics. 2009;25: 1923–1929. doi:10.1093/bioinformatics/btp358
