# Supplementary material for: New pharmacodynamic parameters linked with ibrutinib responses in chronic lymphocytic leukemia: Prospective study in real-world patients and mathematical modeling
Source: PLoS Med. 2024 Jul 22;21(7):e1004430. doi: 10.1371/journal.pmed.1004430 (PMC11262688; doi:10.1371/journal.pmed.1004430)
Supplement: S6 Table — Values are averaged over 10 runs with different initial guess. Parameters are described in Table 1 (main text) and in S1 File Modeling. For fixed parameters Fout, Fin and μ4, superscripts t and p refer to tHL (transient hyperlymphocytosis) and pHL (prolonged hyperlymphocytosis) groups, respectively, identified by a covariate (see Section 2.2 in S1 File Modeling). Coefficient c(x, y) is the correlation coefficient between x and y computed by Monolix (see section 2.1 in S1 File Modeling). (SD–standard deviation; LL–log-likelihood; d–day; NU—no unit). (PDF) [file pmed.1004430.s007.pdf]

Parameter	Value	SD	Units
Fixed Effects			
F_{out}^{t}	0.001	0.0003	d^{-1}
F_{out}^{p}	0.044	0.009	d^{-1}
F_{in}^{t}	0.99	0.08	cells.d^{-1}
F_{in}^{p}	0.219	0.02	cells.d^{-1}
μ_B	0.023	0.0004	d^{-1}
μ_4^t	7.17	0.37	cells^{-1}.d^{-1}
μ_4^p	9.27	0.53	cells^{-1}.d^{-1}
μ_8	9.21	0.50	cells^{-1}.d^{-1}
μ_{NK}	35.77	2.07	cells^{-1}.d^{-1}
μ_{reg}	211.85	12.63	cells^{-1}.d^{-1}
Random Effects			
$\omega_{F_{out}}$	1.89	0.16	d^{-1}
$\omega_{F_{in}}$	1.70	0.06	cells.d^{-1}
ω_{μ_B}	0.67	0.01	d^{-1}
ω_{μ_4}	0.87	0.05	cells^{-1}.d^{-1}
ω_{μ_8}	0.69	0.06	cells^{-1}.d^{-1}
$\omega_{\mu_{NK}}$	1.0	0.04	cells^{-1}.d^{-1}
$\omega_{\mu_{reg}}$	0.97	0.08	cells^{-1}.d^{-1}
Error Parameters			
a_{BLN}	0.23	0.02	cells
b_{Bbl}	0.67	0.009	NU
b_4	0.36	0.002	NU
b_8	0.38	0.003	NU
b_{NK}	0.56	0.004	NU
b_{reg}	0.60	0.006	NU
Correlations			
$c(F_{out}, F_{in})$	-0.71	0.03	NU
$c(\mu_4, \mu_8)$	0.78	0.03	NU
$c(\mu_4, \mu_{NK})$	0.63	0.04	NU
$c(\mu_4, \mu_{reg})$	0.87	0.01	NU
$c(\mu_8, \mu_{NK})$	0.43	0.07	NU
$c(\mu_8, \mu_{reg})$	0.61	0.06	NU
$c(\mu_{NK}, \mu_{reg})$	0.75	0.02	NU
Goodness to fit			
$-2LL$	285	21	NU

S6 Table: **Parameter values associated with the best fit of individual patient measurements.** Values are averaged over 10 runs with different initial guess. Parameters are described in Table 1 (main text) and in S1 File Modeling. For fixed parameters F_{out}, F_{in} and μ_4, superscripts t and p refer to tHL (transient hyperlymphocytosis) and pHL (prolonged hyperlymphocytosis) groups, respectively, identified by a covariate (see Section 2.2 in S1 File Modeling). Coefficient $c(x, y)$ is the correlation coefficient between x and y computed by Monolix (see section 2.1 in S1 File Modeling). (SD – standard deviation; LL – log-likelihood; d – day; NU - no unit).
