# Supplementary material for: New pharmacodynamic parameters linked with ibrutinib responses in chronic lymphocytic leukemia: Prospective study in real-world patients and mathematical modeling
Source: PLoS Med. 2024 Jul 22;21(7):e1004430. doi: 10.1371/journal.pmed.1004430 (PMC11262688; doi:10.1371/journal.pmed.1004430)
Supplement: S1 Fig — Median of percent change in age (A) and absolute lymphocyte counts (ALCs) (B) (insert: according to cohort 1 and 2) in transient hyperlymphocytosis (tHL) and prolonged hyperlymphocytosis (pHL) groups; each dot represents a patient. (C) Percent of patients in tHL (red) and pHL (blue) groups according to genetic alterations. Del: deletion; IGHV M: mutated immunoglobulin heavy chain variable region genes; IGHV UM: unmutated immunoglobulin heavy chain variable region genes. (PDF) [file pmed.1004430.s008.pdf]

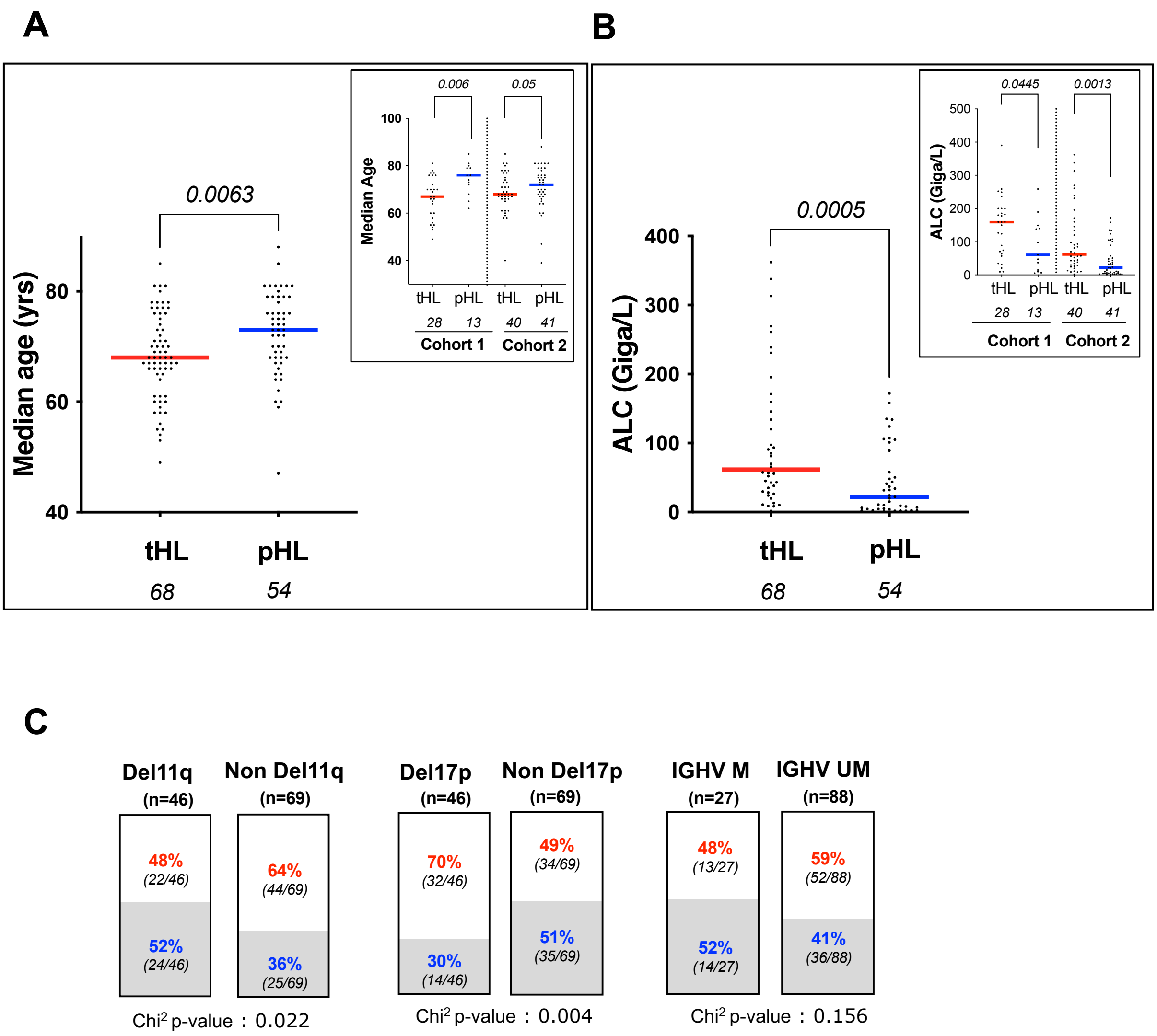

S1 Fig. **Analysis of factors associated with hyperlymphocytosis**. Median of percent change in age **(A)** and absolute lymphocyte counts (ALC) **(B)** (insert: according to cohort 1 and 2) in transient hyperlymphocytosis (tHL) and prolonged hyperlymphocytosis (pHL) groups; each dot represents a patient. **(C)** Percent of patients in tHL (red) and pHL (blue) groups according to genetic alterations. Del: deletion; IGHV M: mutated immunoglobulin heavy chain variable region genes; IGHV UM: unmutated immunoglobulin heavy chain variable region genes.
