# Supplementary material for: New pharmacodynamic parameters linked with ibrutinib responses in chronic lymphocytic leukemia: Prospective study in real-world patients and mathematical modeling
Source: PLoS Med. 2024 Jul 22;21(7):e1004430. doi: 10.1371/journal.pmed.1004430 (PMC11262688; doi:10.1371/journal.pmed.1004430)
Supplement: S2 Fig — Transient hyperlymphocytosis group (tHL) (n = 68); prolonged hyperlymphocytosis group (pHL) (n = 52); each line represents a patient. (PDF) [file pmed.1004430.s009.pdf]

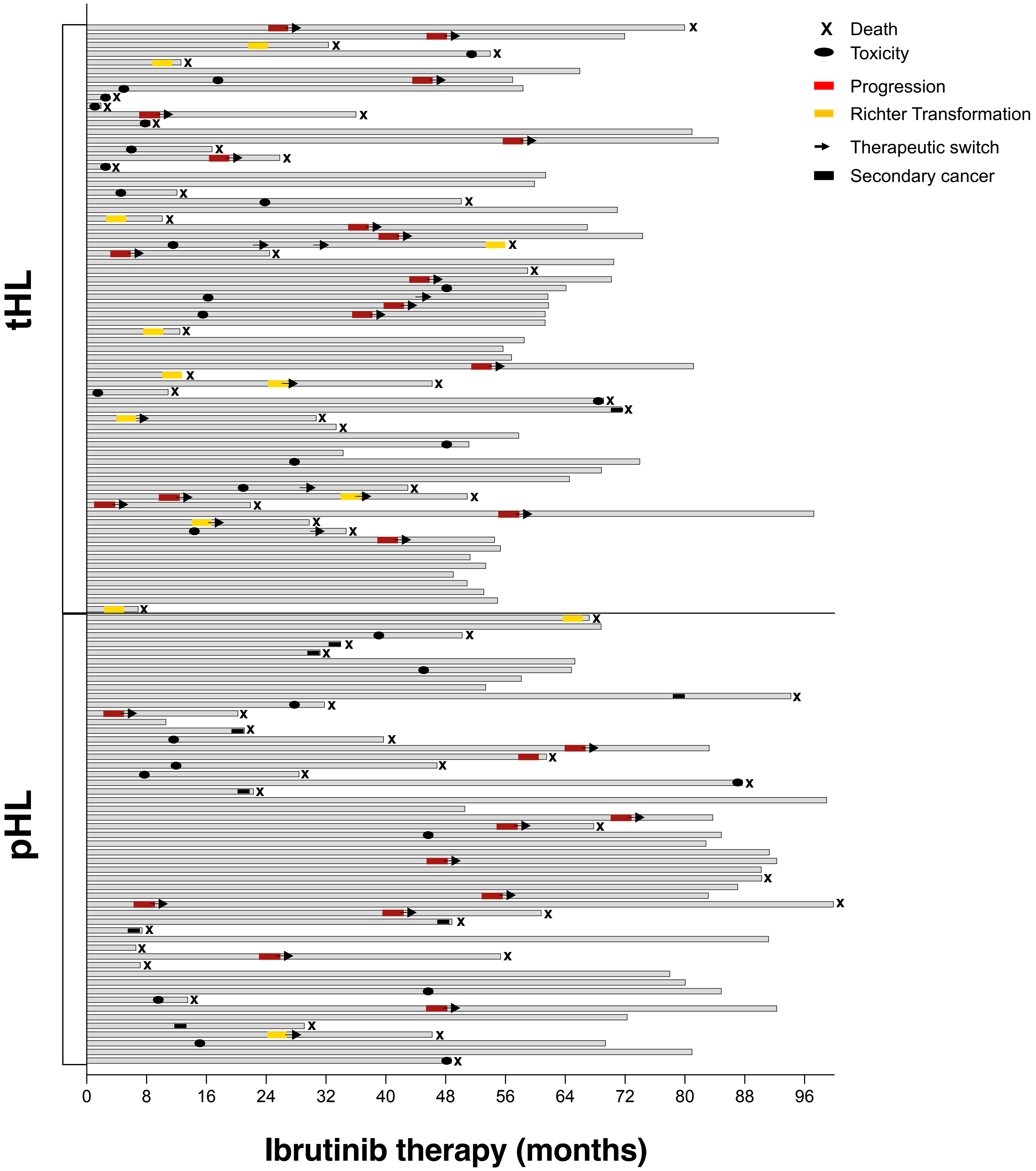

S2 Fig. **Long-term evolution of patients under ibrutinib therapy.** transient hyperlymphocytosis group (tHL) (n=68); prolonged hyperlymphocytosis group (pHL) (n=52); each line represents a patient.
