# Supplementary material for: New pharmacodynamic parameters linked with ibrutinib responses in chronic lymphocytic leukemia: Prospective study in real-world patients and mathematical modeling
Source: PLoS Med. 2024 Jul 22;21(7):e1004430. doi: 10.1371/journal.pmed.1004430 (PMC11262688; doi:10.1371/journal.pmed.1004430)
Supplement: S3 Fig — (A) Representative features of total body MRI for a patient before ibrutinib treatment. Arbitrary colors indicate the different organs of interest (cervical, axillary, mediastinal, retroperitoneal, iliac lymph nodes, spleen, and liver). (B) Volume of lymph nodes according to transient hyperlymphocytosis group (tHL) and prolonged hyperlymphocytosis group (pHL). (C) ADC of lymph nodes according to tHL and pHL groups. SD, standard deviation. (PDF) [file pmed.1004430.s010.pdf]

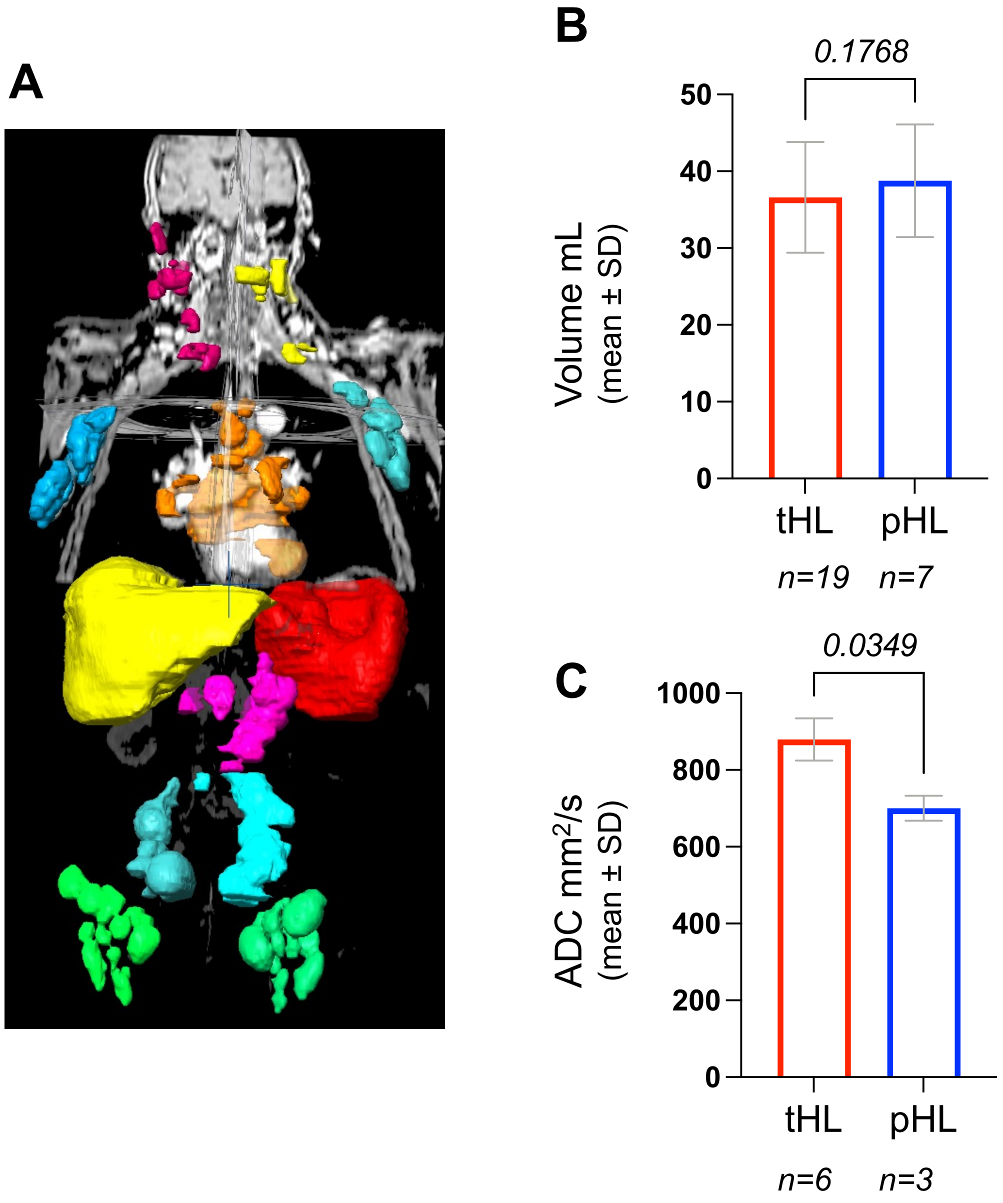

S3 Fig. **Magnetic resonance imaging (MRI) analysis before ibrutinib treatment**. **(A)** Representative features of total body MRI for a patient before ibrutinib treatment. Arbitrary colors indicate the different organs of interest (cervical, axillary, mediastinal, retroperitoneal, iliac lymph nodes, spleen and liver). **(B)** Volume of lymph nodes according to transient hyperlymphocytosis group (tHL) and prolonged hyperlymphocytosis group (pHL); **(C)** ADC of lymph nodes according to tHL and pHL groups. SD: standard deviation
