# Supplementary material for: New pharmacodynamic parameters linked with ibrutinib responses in chronic lymphocytic leukemia: Prospective study in real-world patients and mathematical modeling
Source: PLoS Med. 2024 Jul 22;21(7):e1004430. doi: 10.1371/journal.pmed.1004430 (PMC11262688; doi:10.1371/journal.pmed.1004430)
Supplement: S4 Fig — tHL, transient hyperlymphocytosis group; pHL, prolonged hyperlymphocytosis group; SD, standard deviation; a.u., arbitrary units. (PDF) [file pmed.1004430.s011.pdf]

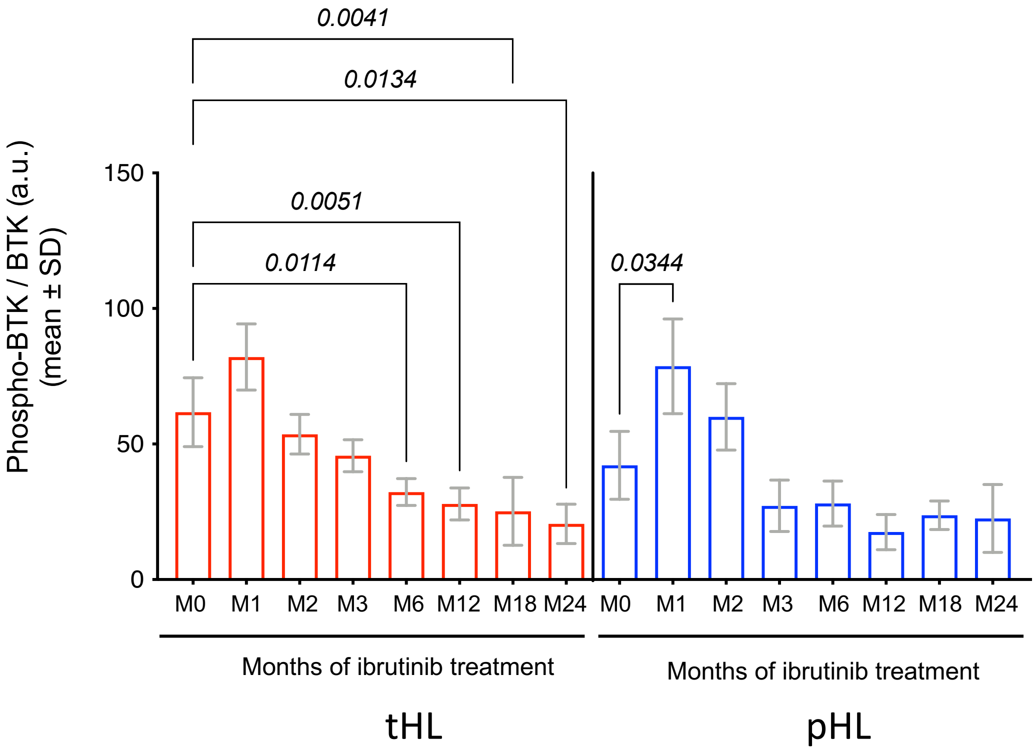

S4 Fig. **Monitoring of phosphoBTK/BTK along ibrutinib treatment**.
tHL: transient hyperlymphocytosis group; pHL: prolonged hyperlymphocytosis group.
SD: standard deviation; a.u.: arbitrary units
