# Supplementary material for: New pharmacodynamic parameters linked with ibrutinib responses in chronic lymphocytic leukemia: Prospective study in real-world patients and mathematical modeling
Source: PLoS Med. 2024 Jul 22;21(7):e1004430. doi: 10.1371/journal.pmed.1004430 (PMC11262688; doi:10.1371/journal.pmed.1004430)
Supplement: S1 File Modeling — Details on modeling choices, parameter estimation methodology, model selection procedure, for both the generic model and the patient-specific model. (PDF) [file pmed.1004430.s012.pdf]

New pharmacodynamic parameters linked with ibrutinib responses: prospective study in real-world patients and mathematical modeling

Sarah Cadot*, Chloe Audebert*, Charlotte Dion, Soleakhena Ken, Loic Dupré, Laetitia Largeaud, Camille Laurent, Loic Ysebaert, Fabien Crauste and Anne Quillet-Mary

Supporting Information: S1 Modeling

1 Mathematical model of an average patient

1.1 Mathematical model.

In order to build a dynamical model of cells dynamics in patients treated with Ibrutinib, we account for clinical observable variables. These variables are leukemic B cell counts in lymph nodes (denoted by B_{LN}), and leukemic B cell (B_{bl}), CD4 T cell (T_4), CD8 T cell (T_8), NK cell (T_{NK}), and Regulatory CD4 T cell (T_{regs}) counts in blood.

Inspired by [9], dynamics of B cells are assumed to be described by

$$\frac{dB_{LN}}{dt} = -\mu_B B_{LN} - F_{out} B_{LN} + F_{in}, \quad (1)$$

$$\frac{dB_{bl}}{dt} = -\mu_B B_{bl} + F_{out} B_{LN}. \quad (2)$$

Leukemic B cells exit the lymph nodes (LN) with a rate F_{out}, and they are produced within LN with a constant rate F_{in}. For the sake of simplicity, the same death rate (μ_B) is assumed for leukemic B cells whether in LN or in blood.

Dynamics of T cells are assumed to follow a standard equation,

$$\frac{dT_X}{dt} = r_X(T_X, B)T_X + F_{in}^X, \quad (3)$$

where r_X is a net growth rate, incorporating cell divisions and deaths, and F_{in}^X a source term of T cells of type X exiting the LN and entering blood.

Different assumptions lead to various renewal rates: whether accounting for proliferation of T cells or not, for Tregs-mediated regulation or not, for B cell-mediated proliferation or not, for instance. Based on the ability to generate dynamics in agreement with measured cell counts, we assumed no proliferation of T cells in blood and a Tregs-mediated regulation of T cell death, that is

$$r_X(T_X, B) = -\mu_X T_{regs}. \quad (4)$$

The specificity of some dynamics observed in the cohort 1 data relies in important, and sometimes prolonged, increase of B or T cell counts in blood following the onset of the treatment. Ibrutinib is known to deplete LN of leukemic B cells, therefore it may be hypothesized that not only B but also T cells exit the LN following Ibrutinib treatment. Ideally, the source term F_{in}^{X} would depend on the number of T cells of type X in LN. Yet, this information is not available, only leukemic B cell counts are measured in LN. We then assumed that for each T cell population the flux of cells from the LN is proportional to the number of leukemic B cells in the lymph nodes, B_{LN}, and write

$$F_{in}^{X} = \alpha_{in}^{X} B_{LN}. \tag{5}$$

The model made of equations (1) to (5) describes the evoluton of absolute B and T cell counts in LN and blood. Since hyperlymphocytosis is defined with respect to the pre-treatment measurement ($t =$ M0), we chose to focus on the evolution of normalized cell counts and rewrote the model,

$$\begin{aligned} \frac{dB_{LN}^{n}}{dt} &= -\mu_B B_{LN}^{n} - F_{out} B_{LN}^{n} + F_{in} \frac{1}{B_{LN}^{0}}, \\ \frac{dB_{bl}^{n}}{dt} &= -\mu_B B_{bl}^{n} + F_{out} \left(\frac{B_{LN}^{0}}{B_{bl}^{0}} \right) B_{LN}^{n}, \\ \frac{dT_{X}^{n}}{dt} &= \alpha_{in}^{X} \left(\frac{B_{LN}^{0}}{T_{X}^{0}} \right) B_{LN}^{n} - \mu_X T_{regs}^{0} T_{regs}^{n} T_{X}^{n}. \end{aligned} \tag{6}$$

where B_{LN}^{n} and B_{bl}^{n} are the normalized cell counts of leukemic B cells in the LN and blood respectively, and T_{X}^{n} the normalized cells counts of T cells of type X. Initial cell counts measured before treatment are denoted by B_{LN}^{0}, B_{bl}^{0} and T_{X}^{0} for leukemic B cells in LN, in blood and for T cells. These initial counts are known from cohort 1 clinical measurements. As a consequence, initial conditions of System (6) equal 1 for all populations.

Preliminary analysis of cell count measurements highlighted a strong correlation between CD8 and CD4 T cell counts ($r^2 = 0.98$ for tHL group , $r^2 = 0.95$ for pHL group). Consequently, in the rest of the modeling part we only considered one equation – for CD4 T cells – instead of two equations for CD8 and CD4 T cell populations. Keeping in mind that there are then 3 types of T cells (CD4, NK and regulatory T cells), System (6) comprises 9 parameters: 3 parameters associated with B cell dynamics and 6 parameters associated with T cell dynamics.

1.2 Data Fitting and Quality-of-Fit Criterion.

Model (6) has been compared to data consisting in mean values of cell counts at M1, M2, M3, M6, M12, M18 and M24 for blood measurements, and M1, M12 an M24 for LN measurements. Least-squares have been used to optimize parameter values, that is the quantity

$$LS_{B_{LN}} + LS_{B_{bl}} + LS_{T_4} + LS_{T_{NK}} + LS_{T_{regs}}$$

has been minimized, where

$$LS_X = \sum_{i=1}^{n_X} \left(X(t_i) - \bar{X}_i \right)^2,$$

with $\bar{X}_i$ the mean value of observable X at time t_i. Number of measurements n_X equals 7 for all cell populations, except for leukemic B cell counts in LN where $n_{B_{LN}} = 3$ (M1, M12, M24).

Parameter value estimation and data fitting have been performed using Data2Dynamics, a Matlab R2019b add-on that allows to fit ordinary differential equation models to data and imple-ments, among other specificities, statistical assessment of parameter [6, 7].

1.3 Model selection.

Model selection was performed from the model made of equations (6). A list of models was compared to data, parameter values were estimated, and statistical indicators computed to determine which model fits data the best.

The list of models is presented in Table 1. All models are modifications of the model made of equations (6) and comprising 9 parameters. Modifications mostly lead to reduce the number of parameters to estimate (from 9 parameters for the initial model down to 5 parameters for the most simplified one). Parameter estimation is performed through the procedure in Section 1.2. In order to balance the quality of fit and the complexity of the models, statistical indicators are used to weigh the ability of a given model to appropriately fit the data. The corrected Akaike Information Criterion (AICc) is used [2]. It is the most adapted criterion here, due to the number of parameters and the quantity of data. However, for information purposes, the Bayesian Information Criterion (BIC) is also computed. It is noticeable that both AICc and BIC provide here the same conclusions.

We have to mention that the B cell model (from Wodarz et al [9]) has not been modified, because it has already been validated in the case of CLL and it very well describes B cell dynamics, in the LN and in blood, as confirmed by our numerous simulations.

We remind that

$$AICc = 2k – 2LL + 2\frac{k(k+1)}{n-(k+1)},$$

with n the size of the sample (here $n = 35$), k the number of parameters, and LL the log-likelihood, and

$$BIC = \log(n)k – 2LL.$$

One may note that the total number of parameters k is equal to the number of parameters of the structural model (here from 5 to 9) plus the 3 error parameters. Indeed, we assumed an error parameter for B cell counts in LN, another error parameter for B cell counts in blood, and a unique error parameter for T cell counts in blood. We performed several tests and none showed any relevance to consider an error parameter for each T cell population.

Noticeably, some models may be unidentifiable. This means that comparison of the model to data does not allow to estimate uniquely parameter values (often combinations of parameter values) that best reproduce the data. Unidentifiability is either due to a lack of information (not enough data to estimate parameter values) or to correlations in parameter values. Here the latter explanation is the reason for unidentifiability (because for most models identifiability is reached, so the problem does not come from the data). We hence indicated in Table 2 when models were identifiable ('y' for identifiable, 'n' for unidentifiable, column 6 labeled 'Ident.').

Results of model selection are presented in Table 2. Depending on the group (tHL or pHL), models fit differently the data, yet model 26 is always associated with the best AICc. This model has then been selected and used throughout the manuscript.

For the sake of clarity, it may be mentioned that other models were tested:

- Variations of models listed in Table 1 with one or more α_{in}^X coefficients equal to zero (meaning no source term for one or more T cell populations) have been compared to data but they generated either unidentifiable models or unsatisfactory models, the selection procedure highlighting that small values of parameters α_{in}^X always gave better results than no parameter α_{in}^X.

- Models accounting for proliferation of T cells (either a constant proliferation rate or a T cell mediated proliferation rate) have also been tested, but fits to data are not better and most of the time models are unidentifiable (due to a lack of information in the data that would allow to measure T cell proliferation).

Table 1: List of models tested. Models are numbered from 1 to 26, and appropriate descriptions are provided.

Model	Description
1	Model (6)
2	Model (6) with $\mu_4 = \mu_{NK}$
3	Model (6) with $\mu_4 = \mu_{NK}$ and $\alpha_{in}^4 = \alpha_{in}^{NK}$
4	Model (6) with $\mu_4 = \mu_{NK}$ and $\alpha_{in}^4 = \alpha_{in}^{reg}$
5	Model (6) with $\mu_4 = \mu_{NK}$ and $\alpha_{in}^{NK} = \alpha_{in}^{reg}$
6	Model (6) with $\mu_4 = \mu_{NK}$ and $\alpha_{in}^4 = \alpha_{in}^{NK} = \alpha_{in}^{reg}$
7	Model (6) with $\mu_4 = \mu_{reg}$
8	Model (6) with $\mu_4 = \mu_{reg}$ and $\alpha_{in}^4 = \alpha_{in}^{NK}$
9	Model (6) with $\mu_4 = \mu_{reg}$ and $\alpha_{in}^4 = \alpha_{in}^{reg}$
10	Model (6) with $\mu_4 = \mu_{reg}$ and $\alpha_{in}^{NK} = \alpha_{in}^{reg}$
11	Model (6) with $\mu_4 = \mu_{reg}$ and $\alpha_{in}^4 = \alpha_{in}^{NK} = \alpha_{in}^{reg}$
12	Model (6) with $\mu_{NK} = \mu_{reg}$
13	Model (6) with $\mu_{NK} = \mu_{reg}$ and $\alpha_{in}^4 = \alpha_{in}^{NK}$
14	Model (6) with $\mu_{NK} = \mu_{reg}$ and $\alpha_{in}^4 = \alpha_{in}^{reg}$
15	Model (6) with $\mu_{NK} = \mu_{reg}$ and $\alpha_{in}^{NK} = \alpha_{in}^{reg}$
16	Model (6) with $\mu_{NK} = \mu_{reg}$ and $\alpha_{in}^4 = \alpha_{in}^{NK} = \alpha_{in}^{reg}$
17	Model (6) with $\mu_4 = \mu_{NK} = \mu_{reg}$
18	Model (6) with $\mu_4 = \mu_{NK} = \mu_{reg}$ and $\alpha_{in}^4 = \alpha_{in}^{NK}$
19	Model (6) with $\mu_4 = \mu_{NK} = \mu_{reg}$ and $\alpha_{in}^4 = \alpha_{in}^{reg}$
20	Model (6) with $\mu_4 = \mu_{NK} = \mu_{reg}$ and $\alpha_{in}^{NK} = \alpha_{in}^{reg}$
21	Model (6) with $\mu_4 = \mu_{NK} = \mu_{reg}$ and $\alpha_{in}^4 = \alpha_{in}^{NK} = \alpha_{in}^{reg}$
22	Model (6) with $\alpha_{in}^4 = \alpha_{in}^{NK}$
23	Model (6) with $\alpha_{in}^4 = \alpha_{in}^{reg}$
24	Model (6) with $\alpha_{in}^{NK} = \alpha_{in}^{reg}$
25	Model (6) with $\alpha_{in}^4 = \alpha_{in}^{NK} = \alpha_{in}^{reg}$
26	Model (6) with $\alpha_{in}^4 = \alpha_{in}^{NK} = \alpha_{in}^{reg} = F_{out}$

Table 2: Models' ranking based on the AICc. For each group (tHL group, left; pHL group, right), models have been ranked based on their AICc values, from the lowest to the higher. Unidentifiable models are ranked after all identifiable models. Column1: model's number; Column 2: number of parameters (par.); Column 3: value of −2 the log-likelihood ($-2LL$); Column 4: AICc value; Column 5: BIC value; Column 6: indicates whether the model is identifiable (y - yes; n - no).

**tHL group**						**pHL group**					
Model	par.	$-2LL$	AICc	BIC	Ident.	Model	par.	$-2LL$	AICc	BIC	Ident.
26	6	−131	−106	−99	y	26	6	−27	−2	5	y
17	7	−134	−105	−98	y	4	7	−26	3	10	y
10	7	−132	−103	−96	y	5	7	−26	3	10	y
5	7	−131	−102	−95	y	10	7	−26	3	10	y
8	7	−131	−102	−95	y	13	7	−26	3	10	y
12	8	−135	−102	−96	y	14	7	−26	3	10	y
2	8	−134	−101	−95	y	25	7	−26	3	10	y
4	7	−130	−101	−94	y	23	8	−25	8	14	y
13	7	−130	−101	−94	y	24	8	−25	8	14	y
25	7	−130	−101	−94	y	19	6	−0	25	32	y
7	8	−132	−99	−93	y	9	7	−2	27	34	y
22	8	−130	−97	−91	y	6	6	7	32	39	y
1	9	−134	−96	−91	y	11	6	7	32	39	y
18	6	−111	−86	−79	y	21	5	16	38	44	y
6	6	−108	−83	−76	y	20	6	16	41	48	y
3	7	−110	−81	−74	y	15	7	17	46	53	y
9	7	−107	−78	−71	y	1	9	−24	-	-	n
21	5	−100	−77	−73	y	2	8	−25	-	-	n
11	6	−104	-	-	n	3	7	−7	-	-	n
14	7	−130	-	-	n	7	8	−25	-	-	n
15	7	−104	-	-	n	8	7	−26	-	-	n
16	6	−99	-	-	n	12	8	−25	-	-	n
19	6	−107	-	-	n	16	6	17	-	-	n
20	6	−99	-	-	n	17	7	−26	-	-	n
23	8	−130	-	-	n	18	6	−8	-	-	n
24	8	−131	-	-	n	22	8	−25	-	-	n

- Finally, models that do not consider regulation of T cell dynamics by Tregs have also been considered, but they result in poor reproduction of T cell dynamics.

2 Population approach and inter-patient variability

After validating a model of B and T cell dynamics under Ibrutinib treatment for an average patient, we modified the model to account for inter-patient variability. To do so, we used a population approach based on mixed-effect modeling [4].

2.1 Nonlinear mixed-effect models

Nonlinear mixed effects models allow the description of inter-patient variability within a population of individuals. All individuals belong to the same population (here a population of LLC patients) so they share common characteristics. These are called "fixed effects" and characterize an average behavior of the population. Nevertheless, each patient is unique and differs from the average behavior by a specific value called "random effect". Details on the method can be found in [1, 3, 4, 8], we here describe only the part relevant to this study, focusing on our hypotheses.

Data $\{d_{i,j},\, i = 1, ..., N_{ind},\, j = 1, ..., n_i\}$ is assumed to satisfy

$$d_{i,j} = f(y_{i,j}, \psi_i) + (a + bf(y_{i,j}, \psi_i))\varepsilon_{i,j},$$

where $d_{i,j}$ is the j-th observation of patient i, N_{ind} is the number of patients within the population and n_i is the number of observations for the i-th patient.

The function f accounts for individual dynamics generated by a mathematical model. In this work f is associated with the solution of a system of ODE, see Section 1.1. The function f depends on known variables, denoted by $y_{i,j}$ (*e.g* $B^0_{LN,i}$, the initial ($j = 0$) count of leukemic B cells in LN for patient i), and parameters of the i-th patient, denoted by ψ_i.

Patient-specific parameter vector ψ_i is assumed to be split into fixed effects (population-dependent effects, average behavior) and random effects (patient-dependent effects). In addition, parameters ψ_i are assumed to follow a log-normal distribution to ensure their positivity. If ψ_i^k denotes the k-th parameter characterizing patient i, then it is assumed that

$$\log(\psi_i^k) = \log(p^k_{pop}) + \eta_i^k,$$

where the vector of parameters $p_{pop} = (p^k_{pop})_k$ models the average behavior of the population, and $\eta_i = (\eta_i^k)_k$ represents how patient i differs from this average behavior. Random effects follow a normal distribution : $(\eta_i^k)_k \sim \mathcal{N}(0, \Omega)$. Ω is the variance-covariance matrix defining the distribution of the vector of random effects. When no correlation between random effects is considered, Ω is a diagonal matrix with coefficients ω_k^2 in the diagonal. The coefficients ω_k^2 quantify the variability of the k-th parameter within the population. When a correlation between random effects is considered, the variance-covariance matrix Ω is a block diagonal matrix, with $\Omega_{i,j} = \Omega_{j,i} = c(\eta_i, \eta_j)\omega_i\omega_j$, where $c(\eta_i, \eta_j)$ is the correlation coefficient between random effects η_i and η_j.

The residual errors, combining model approximations and measurement noise, are denoted by $(a + bf)\varepsilon_{i,j}$. They quantify how the model prediction is close to the observation. Residual errors are assumed independent, identically and normally distributed, *i.e* $\varepsilon_{i,j} \sim \mathcal{N}(0, 1)$. Moreover, the random effects η_i and the residual errors $(a + bf)\varepsilon_{i,j}$ are mutually independent.

2.2 Parameter estimation

Parameter values are estimated with Stochastic Approximation Expectation-Maximization (SAEM) algorithm. The SAEM algorithm is available in [5].

2.2.1 Population and patient-specific parameters.

Under the previous assumptions, cell population dynamics (average behavior and inter-patient variability) are described by parameters: p_{pop}, Ω, and a or b. These parameters are estimated by likelihood maximization with the SAEM algorithm.

Once these parameters have been estimated, each patient-specific vector of parameters ψ_i is estimated by maximizing the conditional probabilities

$$\mathbb{P}(\psi_i | d_{i,j}; \hat{p}_{pop}, \hat{\Omega}, \hat{a}, \hat{b}),$$

where $\hat{x}$ denotes the estimated value of x.

2.2.2 Covariates.

In order to characterize patients from tHL and pHL groups, we used categorical covariates. To do so, data of tHL and pHL groups have been pooled together, then parameter values have been estimated (see paragraph above) by assuming that fixed effects parameters of patients from pHL group were different from fixed effects parameters of tHL group patients.

Categorical covariates were introduced as follows: if a patient is either in tHL or pHL group, we assume that the probability distribution of their patient-specific parameter vector ψ_i has a different mean. We write

$$\log(\psi_i^k) = \log(p_{pop}^k) + \beta^k c_i + \eta_i^k,$$

where c_i equals 1 if patient i is in pHL group, and 0 if patient i is in tHL group, and $\beta = (\beta^k)_k$ is a vector of covariate parameters. Estimated covariate parameters $\hat{\beta}$ have been tested to be significantly different from zero with a Wald test implemented in Monolix software [5], with a p-value threshold at 0.05.

Patient dynamics are then characterized by parameters $(p_{pop}, \Omega, a, b, \beta)$. If the estimated vector $\hat{\beta}$ is significantly different from zero, then the classification in tHL or pHL groups partly explains the observed variability.

References

[1] Delyon B, Lavielle M, Moulines E (1999) Convergence of a stochastic approximation version of the EM algorithm. The Annals of Stat 27(1), 94–128.

[2] Hurvich CM and Tsai C-L (1989) Regression and time series model selection in small samples. Biometrika 76, 297—307.

[3] Kuhn E, Lavielle M (2005) Maximum likelihood estimation in nonlinear mixed effects models. Computational Statistics and Data Analysis 49(4), 1020–1038.

[4] Lavielle M (2014) Mixed effects models for the population approach. Models, Tasks, Methods and Tools. Chapmann and Hall/CRC , 383p.

[5] Monolix version 2019R1. Antony, France: Lixoft SAS, 2019.

[6] Raue A., et al. (2015) Data2Dynamics: a modeling environment tailored to parameter estimation in dynamical systems. Bioinformatics, 31(21), 3558-3560.

[7] Raue A., et al. (2013) Lessons Learned from Quantitative Dynamical Modeling in Systems Biology. PLOS ONE, 8(9), e74335.

[8] Samson A, Donnet S (2007) Estimation of parameters in incomplete data models defined by dynamical systems. Journal of Statistical Planning and Inference 137 (9), 2815–2831.

[9] Wodarz D, Garg N, Komarova NL, Benjamini O, Keating MJ, Wierda WG, Kantarjian H, James D, O'Brien S, Burger JA (2014) Kinetics of CLL cells in tissues and blood during therapy with the BTK inhibitor ibrutinib. Blood 123(26), 4132–4135.
